# Anti-cancer potentiality of linoelaidic acid isolated from marine Tapra fish oil (*Ophisthopterus tardoore*) via ROS generation and caspase activation on MCF-7 cell line

**DOI:** 10.1038/s41598-023-34885-3

**Published:** 2023-08-29

**Authors:** Ananya Dutta, Titli Panchali, Amina Khatun, Sreenivasa Rao Jarapala, Koushik Das, Kuntal Ghosh, Sudipta Chakrabarti, Shrabani Pradhan

**Affiliations:** 1 Department of Paramedical and Allied Health Sciences, Midnapore City College, Midnapore, 721129 West Bengal India; 2 Department of Food Chemistry and Nutrient Analysis, National Institute of Nutrition (NIN), Hyderabad, Telengana 500007 India; 3 Department of Nutrition, Belda College, Paschim Medinipur, 721424 West Bengal India

**Keywords:** Cancer, Breast cancer

## Abstract

The implication of inflammation in the pathophysiology of several types of cancers has been under intense investigation. Conjugated fatty acids can modulate inflammation and present anticancer effects, promoting cancer cell death. In this paper, we evaluated the efficacy of new conjugated fatty acids isolated from marine *Opisthopterus tardoore* (Tapra fish) in human breast cancer cell lines MCF-7. Linoelaidic acid, a marine fish (*O. tardoore*) derived unsaturated fatty acids, showed effective anticancer activity against MCF-7. Cell viability (MTT) assay revealed a dose-dependent decline in cancer cell viability. It was noteworthy that 5 µM linoelaidic acid decreased the MCF-7 cell viability by 81.82%. Besides that, linoelaidic acid significantly (P< 0.05) increased the level of tumor necrosis factor-α (TNF-α) and interleukin-1 receptor antagonist (IL-1ra) studied by ELISA. Not only that, linoelaidic acid significantly decreased the reduced glutathione level and increased the oxidized glutathione level in MCF-7 cells indicating the oxidative stress inside the cell. Two different cell staining methods with acridine orange-ethidium bromide and DAPI confirmed that the linoelaidic acid rendered their detrimental effect on cancer cells. To decipher the mode of apoptosis Western blotting was performed in which the expression pattern of several proteins (p53, IL-10, and IL-1ra) established the apoptosis in the studied cell lines after linoelaidic acid exposure. Hence it may be conferred that linoelaidic acid has prompt anticancer activity. Therefore this drug can be used further for the treatment of cancer.

## Introduction

Breast cancer, a heterogeneous disease with diversity in morphological features and histological characteristics, is a major global health problem in women^1^. Breast cancer is the second leading cause of cancer-related deaths occurs in female between the ages of 40 and 50 years in industrialized nations. The lifetime risk of breast cancer prognosis in women is higher than for any other malignancy. Although the predominant driving force of breast carcinogenesis is thought to be hormonal, cytokine production and inflammation are also being recognized as important crucial factor for cancer progression and development. Breast cancer claimed the lives of 2.08 million people out of 18.08 million new cancer cases (an 11.6% incidence rate) and 626,679 people out of 9.55 million cancer-related deaths (6.6% of all cancer-related fatalities) worldwide in 2018 (WHO, 2021). Breast cancer was detected in 162,468 new cases in 2018, accounting for 27.7% of all new malignancies among Indian women and 11.1% of all cancer fatalities^2^. The incidence of breast cancer in the Indian population was significantly differed in comparison to the western population. The frequency of this incidence is highly proportional to the Indian patients at the premenopausal stage and the maximum age between 40 to 50 years^3, 4^. Most of the chemotherapeutic agents used to treat this life-threatening disease are highly toxic with long-term side effects. Therefore, novel establishment of anti-cancer drugs with higher efficiency and specificity are urgently needed. Marine fishes are becoming well-known sources of fatty acids as described in our previous report as well as several other reports^5, 6^. Marine fishes are reservoirs of essential nutrient elements such as amino acids (lysine, S-containing amino acids), and long-chain omega-3 polyunsaturated fatty acids (eicosapentaenoic acid, docosahexaenoic acid), vitamins (A, D) and trace elements which are required for human health. Several investigations had already proved that omega 3 and 6 fatty acids such as docosahexaenoic acid (DHA), eicosapentaenoic acid (EPA), and arachidonic acid (AA) exert anticancer activities by inducing apoptosis, inhibiting cell proliferation, suppressing of neoplastic transformation, and anti-angiogenicity^7^. Typically, fish oil inhibited the growth of cancer cells by suppressing integrin-linked kinases^8^. The anti-inflammatory activity of fish oil was demonstrated by significantly reduced expression of pro-inflammatory genes or related products, such as leukotriene B4 (LTB4), phosphoinositide 3-kinase (PI3Kα), interleukin-1β (IL-1β), interleukin-10 (IL-10), and interleukin-23 (IL-23) in the peripheral blood mononuclear cells (PBMC) of a healthy population given fish oil-derived EPA (775 mg/day) for 5 weeks^9^. Up to date, no established chemopreventive drugs found in the market without side effects and these are not cost effective. Studies of the effect of linoelaidic acid derived from marine Tapra fish (*Opisthopterus tardoore*) have not been reported yet for induction of apoptosis in various cancer cell lines. Early studies already reported omega-3 fatty acids shown anticancer activity by inhibiting cell growth as well as executing cell death^10^. Zheng et al. conducted a comprehensive meta-analysis of 21 independent prospective cohort studies and proved that dietary intake of marine n-3 polyunsaturated fatty acids (PUFAs) was associated with a 14% risk reduction of breast cancer^11^. Another reports also confirmed that n-3 PUFA intake is linked with a reduced risk of breast cancer cell as shown in vivo studies with breast cancer cell lines (MCF-7, KPL-1, MDA-MB-231) or in a case–control study^12, 13^. Further exploration of new connections of linoelaidic acid with other chemotherapy or targeted agents could be considered. In this study, we applied the linoelaidic acid on MCF-7 cell line to evaluate the anticancer activities. Moreover, we proved that linoelaidic acid induced MCF-7 cell death via p53 and caspase activation.

## Materials and methods

### Materials

The human breast cancer cell line (MCF-7) was obtained from the National Centre for Cell Science (NCCS), Pune, India. The heat-inactivated fetal bovine serum (FBS), Phosphate Buffered Saline (PBS) solution (10X), Doxorubicin (DOX), Penicillin–streptomycin solution, and Trypsin–EDTA 10X-solution were purchased from HiMedia (Maharashtra, India).

Both the cell viability kit for 3-(4,5-dimethylthiazol-2-yl)-2,5-diphenyl tetrazolium bromide (MTT) assay and Cytotoxicity Detection Kit for lactate dehydrogenase (LDH) were purchased from Hi-Media Cell Culture. Linoelaidic acid purchased from Sigma Aldrich (cat# 56769-1ML). Mouse ELISA kit for TNF-α and IL-1ra was obtained from Wuhan Fine Biotech (Wuhan, China).The primary antibodies: p53 mouse monoclonal IgG (Sc-126), Caspase-3 mouse monoclonal IgG (Sc-7272), Caspase-9 mouse monoclonal IgG (Sc-56076), Bax mouse monoclonal IgG (Sc-7480) were purchased from Santa Cruz Biotechnology (Santa Cruz, CA-USA) and IL-10 rabbit monoclonal IgG (cat# DF6894), IL-1ra rabbit monoclonal IgG (cat# DF6812) were purchased from Affinity Biosciences (USA). The secondary antibodies: goat anti-rabbit IgG-HRP (cat# 11-315) and goat anti-mouse IgG-HRP (cat# 11-301) were purchased from Abgenex (Odisha, India).

The primers: **GAPDH** (sense: GGTGAAGGTCGGAGTCAACG, antisense: GTGAAGACGCCAGTGGACTC), **TNF-α** primer set (sense: TTCTGTCTACTGAACTTCGGGGTGATCGGTCC antisense: GTATGAGATAGCAAATCGGCTGACGGTGTGG), **IL-1ra** primer set (sense: GCAGCACAGGCTGGTGAATGAC antisense: TGCCCCCGTGGATGCCCAAG), **IL1r1** primer set (sense: GGTGCCTCTGCTGTCGCTGG antisense: CGCTGTGGGAAGGTGGCCTG), **IL-1β** primer set (sense: ATGGCAACTGTTCCTGAACTCAACT antisense: CAGCACAGGTATAGATTCTTTCCTTT), **INOS** primer set (sense: CCCTTCCGAAGTTTCTGGCAGCAGC antisense: GGCTGTCAGAGCCTCGTGGCTTTGG), **SOCS3** primer set (sense: CGCCTCAAGACCTTCAGCTC antisense: CTGATCCAGGAACTCCCGAA) were purchased from Bioserve, India.

### Methods

#### Thin layer chromatography (TLC)

TLC was carried out on the 20 × 20 cm microcrystalline cellulose-coated plates (Camag, Muttanez, Switzerland). On the plate, a spot of 1 µl of *O. tardoore* fish oil was observed next to the linoelaidic acid. The mobile phase for the one-dimensional TLC analysis was a mixture of methanol, chloroform, and hexane (7:2:1 v/v/v). After spraying the 2′,7′-Dichlorodihydrofluorescein diacetate (H2DCFDA) reagent, spots were seen^14^.

#### Cell treatment

In order to determine the viability of cell, LDH release, and reactive oxygen species (ROS) formation in a dose- and time-dependent manner, cells were seeded in 96-well plates at a density of 1 × 10^6^ cells/ml media. Following this, cells were treated for 24 h with linoelaidic acid at final concentrations of 2 µM or 5 µM and doxorubicin at the concentration of 5 µM treated for 24 h in a humidified (5%) CO_2_ incubator at 37 °C. Cells were seeded in 6-well plate at a density of 1 × l0^6^ cells/ml of media for western blotting and incubated for 24 h at 37 °C in a humidified (5%) CO_2_ incubator before being exposed to doxorubicin and linoelaidic acid at various doses for 24 h. MCF-7 cells underwent 25–30 passages, whereas p53 overexpressing MCF-7 cells underwent 10–12 passages.

MCF-7 cells were divided into the following 4 groups: Group I where only culture media was applied on MCF-7 cell; Group II where MCF-7 cell was treated with 5 µM doxorubicin in culture media; Group III where MCF-7 cell was treated with 2 µM linoelaidic acid in culture media; Group IV where MCF-7 cell was treated with 5 µM linoelaidic acid in culture media.

#### Cell viability and cytotoxicity assays

MTT Assay. The MTT test kit was used according to the manufacturer's instructions to assess the impact of linoelaidic acid on the viability of MCF-7 cells. Comparing cells treated with linoelaidic acid to control cells, the absorbance of the generated colour (595 nm) was assessed using an ELISA microplate reader. The control's absorbance was regarded as having 100% viability. The ratio of absorbance obtained from treated cells to those in the control group multiplied by 100 was used to calculate the percentage viability. The percentage of proliferation was calculated by using the following equation, % of viable cell = (OD_sample_ − OD_blank_)/(OD_treated_ − OD_blank_) × 100^15^.

LDH release. According to the manufacturer's instruction, LDH released in MCF-7 cells treated with doxorubicin and linoelaidic acid was measured using the Cytotoxicity Detection Kit. Released LDH was combined with an enzyme assay in the culture media to produce a red colour, the intensity of which was assessed at 490 nm using an ELISA microplate reader. According to the manufacturer's instructions, the manufacturer determined the percentage cytotoxicity indicated as a percent release of LDH in relation to controls^16^.

#### Cytokine analysis

According to the manufacturer's instructions, the release of tumor necrosis factor-α (TNF-α) and interleukin-1 receptor antagonist (IL-1ra) into media were quantified using a sandwich enzyme-linked immune-sorbent assay (ELISA) kit (Wuhan Fine Biotech). MCF-7 cells were seeded in 6 well plates (1 × 10^6^ cells/ml) prior to 24 h treatment with DOX and linoelaidic acid. Anti-TNF-α and anti-IL-1ra antibody was pre-coated onto 96-well plates. Briefly, wash plate for two times before adding standard, sample (cell culture supernatant) and control wells. Then, 100 µl standard or sample to each well and incubate for 90 min at 37 °C. Following 2 times washing steps, the standards, test samples and biotin conjugated antibody working solution were added to the wells subsequently, incubated at 37 °C for 60 min and washed thrice with wash buffer. 100 µl HRP-Streptavidin conjugate (SABC) working solution was then added and unbound conjugates were washed away by 5 times washing with wash buffer. Then, 90 µl TMB substrates were added and incubated for 15–30 min in dark to visualize HRP enzymatic reaction. TMB was catalyzed by HRP to produce a blue colour product that changed into yellow after adding 50 µl acidic stop solution. The density of yellow colour is directly proportional to the amount of TNF-α and IL-1ra present in sample captured in plate. The absorbance was read using ELISA reader (Robonik) at 450 nm, and the concentration of TNF-α and IL-1ra was determined relative to standard curve.

#### Total nitric oxide assay (NO)

MCF-7 cells were plated in 96-well plates at a density of 1 × 10^6^ cells/ml and incubated for 24 h at 37 °C with 5% CO_2_. Linoelaidic acid was then applied to the cells for 24 h. The EZAssay TM Nitric Oxide Estimation Kit (CCK061-200) was used to measure the nitric oxide assay after 24 h of treatment^19^.

#### Determination of reduced glutathione (GSH)

The standard approach was used to quantify the glutathione (GSH) estimate in cell lysate (1 × 10^6^ cells/ml). Each sample's cell lysate was generated following the application of linoelaidic acid at various doses. It was then mixed with 25% trichloroacetic acid (TCA) and centrifuged at 2000×*g* for 15 min to remove any precipitated proteins. The supernatant was aspirated and diluted to 1 ml with 0.2 M sodium phosphate buffer (pH 8.0). Then, 2 ml of and 5,5′-dithio-bis(2-nitrobenzoic acid) (DTNB) (0.6 mmol) was added. The optical density of the yellow complex produced by the reaction of GSH and DTNB (Ellman's reagent) was measured at 405 nm after 10 min of mixing. Utilizing regularly reduced glutathione, a standard curve was produced. The amounts of GSH were expressed as µg of GSH/mg protein^17^.

#### Determination of oxidized glutathione (GSSG)

After derivatizing GSH using 2-vinyl pyridine, the glutathione disulfide (GSSG) level was assessed using the prescribed methodology. MCF-7 cells (1 × 10^6^ cells/ml) were treated with linoelaidic acid at two different doses as 2 µm/ml and 5 µm/ml, and then they underwent three rounds of washing before cell lysate was created. Briefly, 0.5 ml of the test sample was added to 2 µl of 2-vinylpyidine, and the mixture was then incubated at 37 °C for 1 h. After deproteinizing the reaction mixture with 4% sulfosalicylic acid, the precipitated proteins were settled by centrifuging the mixture at 1000×*g* for 10 min. The solution's supernatant was collected, and the GSSG level was determined using the DTNB reaction at 412 nm absorption and calculated using a standard GSSG curve. The standard GSSG curve was used to determine the level of GSSG. The levels of GSSG were expressed as μg of GSSG/mg protein^18^.

#### Determination of lipid peroxidation (MDA)

Malondialdehyde (MDA) level measurements were used to estimate lipid peroxidation. In short, cell lysate (1 × 10^6^ cells/ml) of MCF-7 was produced following the treatment schedule with linoelaidic acid at at two different doses as 2 µm/ml and 5 µm/ml and DOX. This cell lysate served as the sample for estimating the MDA level. The lipid peroxidation values were reported as nmol/mg protein^20^.

#### RNA isolation

MCF-7 cells were seeded at a density of 1 × 10^6^ cells/ml of a 6-well plate. After being incubated overnight for cell attachment, the cells were treated with 2 µm/ml and 5 µm/ml of linoelaidic acid for 24 h after being added with serum-free media. Untreated control cells were included. After 24 h the cells were trypsinized and collected by centrifugation at 3000 rpm for 15 min. RNA extraction was carried out following the protocol of the HiPurA Total RNA Miniprep purification kit (HiMedia). Briefly wash the cell with PBS, centrifuge it at 3000 rpm for 5 min, and discard the PBS. 350 µl of a lysis solution with β-Mercaptoethanol at (1000:10) were added into the pellet to lyse the cell and collect the cells by centrifugation at 14,000 rpm for 2 min. Then 1 volume of 70% ethanol was added to the cell lysate and mix vigorously by pipetting. RNA binding was performed by transferring the mixture to HiElute Miniperp Spin Column and centrifuged at > 10,000 rpm for 15 s. Then this column was washed once with 700 μl pre-wash solution and centrifuged at > 10,000 rpm for 1 min, again 500 μl wash solution was added and centrifuged at > 10,000 rpm for 1 min. Dry the membrane and transfer the HiElute Column to a 2 ml collection tube. Next 30–50 μl elution solution was added to the tube and centrifuged at > 10,000 rpm for 1 min to obtain a purified RNA sample. The concentration and quality of RNA were quantified by using a nanophotometer (Eppendorf).

#### Reverse transcription and polymerase chain reaction (PCR)

The Hi-cDNA synthesis kit's instructions were followed to prepare the reverse transcription reaction mixture (HiMedia, HiGenoMB, Maharashtra, India). Briefly 1 μl oligo (dT) was added with RNA template (5 ng to 5 μg) and up to 10 μl molecular biology grade water for PCR. Then incubate for 5 min at 65 °C then cool immediately on ice. For preparing the reaction mixture in a total volume of 20 μl, 10 μl of RNA primer mixture was added with 4 μl RT buffer for MMuLV, 2 μl 10 × solution for MMuLV, 1 μl M-MuLV Reverse Transcriptase, 2 μl 10 mM dNTP mix, and volume up to 20 μl with molecular biology grade water for PCR. The reverse transcription reaction was performed as follows—reverse transcription at 42 °C for 60 min 1 cycle and denaturation at 70 °C for 5 min 1 cycle. Subsequently, the cDNA product was amplified by PCR reaction. The PCR reaction mixture, which consists of 9 μl DPEC water, 9 μl 2X PCR TaqMixture, and 0.5 μl each forward and reverse primer with 1 μl cDNA was prepared. A published housekeeping primer set was used for **GAPDH** (sense: GGTGAAGGTCGGAGTCAACG, antisense: GTGAAGACGCCAGTGGACTC), **TNF-α** primer set (sense: TTCTGTCTACTGAACTTCGGGGTGATCGGTCC antisense: GTATGAGATAGCAAATCGGCTGACGGTGTGG), **IL-1ra** primer set (sense: GCAGCACAGGCTGGTGAATGAC antisense: TGCCCCCGTGGATGCCCAAG), **IL1r1** primer set (sense: GGTGCCTCTGCTGTCGCTGG antisense: CGCTGTGGGAAGGTGGCCTG), **IL-1β** primer set (sense: ATGGCAACTGTTCCTGAACTCAACT antisense: CAGCACAGGTATAGATTCTTTCCTTT), **INOS** primer set (sense: CCCTTCCGAAGTTTCTGGCAGCAGC antisense: GGCTGTCAGAGCCTCGTGGCTTTGG), **SOCS3** primer set was selected (sense: CGCCTCAAGACCTTCAGCTC antisense: CTGATCCAGGAACTCCCGAA). Next, PCR was performed as follows: initial denaturation of DNA at 95 °C for 1 min, denaturation at 95 °C for 30 s, DNA annealing at 65 °C for 45 s, and extension of DNA at 72 °C for 2 min, final extension of DNA at 72 °C for 10 min. the second and third steps were repeated for a total of 40 thermal cycles. Lastly, the PCR tube with the sample was held at 4°C.

#### Gene expression analysis

The GeNei^TM^mini-submarine gel system was used to investigate the multiplex of gene expression in linoelaidic acid-treated MCF-7 cells. The primers of all genes were supplied by Bioserve (Telangana, India). Briefly, 5 μl of PCR product was mixed with 1 μl sample loading dye and 5 μl of DNA size standard 3000. Run using GeNei^TM^mini-submarine gel system (co. name). The amplified fragments were separated according to their respective size by a mini-submarine gel system. Results were analyzed using the Gel Documentation Imaging System (Bio-Rad, Model No. 1708275).

#### Immunoblotting analysis of p53, Caspase 3, 9, IL-10, IL-1ra and Bax

The MCF-7 cells treated with the linoelaidic acid were lysed with radioimmunoprecipitation assay (RIPA) buffer, and the protein content was quantified by the Lowry method. 50 µg protein samples was placed in loading buffer and boiled for 5 min, then electrophoresed by Sodium dodecyl-sulfate polyacrylamide gel electrophoresis (SDS-PAGE) and transferred to a nitrocellulose membrane (Bio-Rad, Model No. 165.8033FC)**.** The membrane was then blocked with 4% bovine serum albumin (BSA) and incubated with primary antibody overnight at 4°C. The primary antibody used in this study included β-actin, p53, IL-10, IL1ra, caspase 3, 9, and Bax at a 1:1000 ratio. The membrane was then washed three times with phosphate buffer saline with tween-20 (PBST) and incubated with horseradish peroxidase (HRP) labelled anti-mouse secondary antibody 1:7500 (Abgenex) for 1 h at 4 °C. Specific protein bands were detected using 3,3′,4,4′-Tetraaminobiphenyl tetrahydrochloride (DAB). When inhibitors were employed, cells were pre-treated for 8 h with inhibitors before the addition of linoelaidic acid^21^. ImageJ software was used to measure protein immunoreactivity.

#### Detection of apoptotic morphological changes by immunohistochemical staining using acridine orange‑ethidium bromide (AO-EtBr) staining

Breast cancer cell lines were cultured at 1 × 10^6^ cells/ml in six-well plates and treated with 2 μM/ml and 5 μM/ml of linoelaidic acid for 24 h. Further, Cells were harvested and stained with AO-EtBr dye mix (1:1 v/v**)** from 100 μg/ml in PBS and studied using a fluorescent microscope (Olympus, Model No. BX43F) according to the standard protocol^22^.

#### ROS levels in linoelaidic acid treated MCF-7 cells

The intracellular ROS concentration was determined using active oxygen sensing. Using a fluorescence microscope, H2DCFDA was deacetylated intracellularly by a non-specific esterase to produce the fluorescent compound 2,7-dichlorofluorescein (DCF). Linoelaidic acid was administered to cells for 24 h at the appropriate concentrations (2 µM/ml and 5 µM/ml). PBS was used to clean the cells before 1 mg/ml of H2DCFDA was applied for 30 min at 37 °C^7^. DCF presence was determined using a fluorescence microscope (Olympus, Model No. BX43F). Quantitative result was obtained by Multimode microplate reader at 480 and 530 nm.

#### Nuclear morphological assessment by DAPI staining

In vitro apoptosis was recognized by 4′,6-diamidino-2-phenylindole (DAPI) staining. Breast cancer cells were seeded at a density of 1 × 10^6^ cells/ml in six-well plates and subjected to 24 h of treatment with 2 µM/ml and 5 µM/ml linoelaidic acid and DOX. Cells were harvested, dyed with the 300 nM DNA-binding dye DAPI and observed under a fluorescence microope^23^.

### Statistical analysis

Statistical analyses were conducted using GraphPad Prism 8.0.1 software (GraphPad Software, La Jolla, CA, USA). Results were expressed as the mean ± standard error of the mean (SEM). Statistical significance was then examined using the One-way Analysis of Variance (ANOVA) followed by Tukey’s multiple-comparison test. P values ˂ 0.05 were considered statistically significant.

## Results

### Presence of linoelaidic acid on extracted *Opisthopterus tardoore* fish oil using thin layer chromatography

The current investigation revealed that linoelaidic acid was present in *Opisthopterus tardoore* fish oil. Our results indicated that the same R_f_ value compared with standard linoelaidic acid confirming the presence of linoelaidic acid in the *O. Tardoore* fish oil (Supplementary Figure). Linoelaidic acid was present in this fish oil, which were easily identified by its distinctive blue/green fluorescence in ultraviolet light.

### Effect of linoelaidic acid on the viability of MCF-7 cells

**Figure 1:**
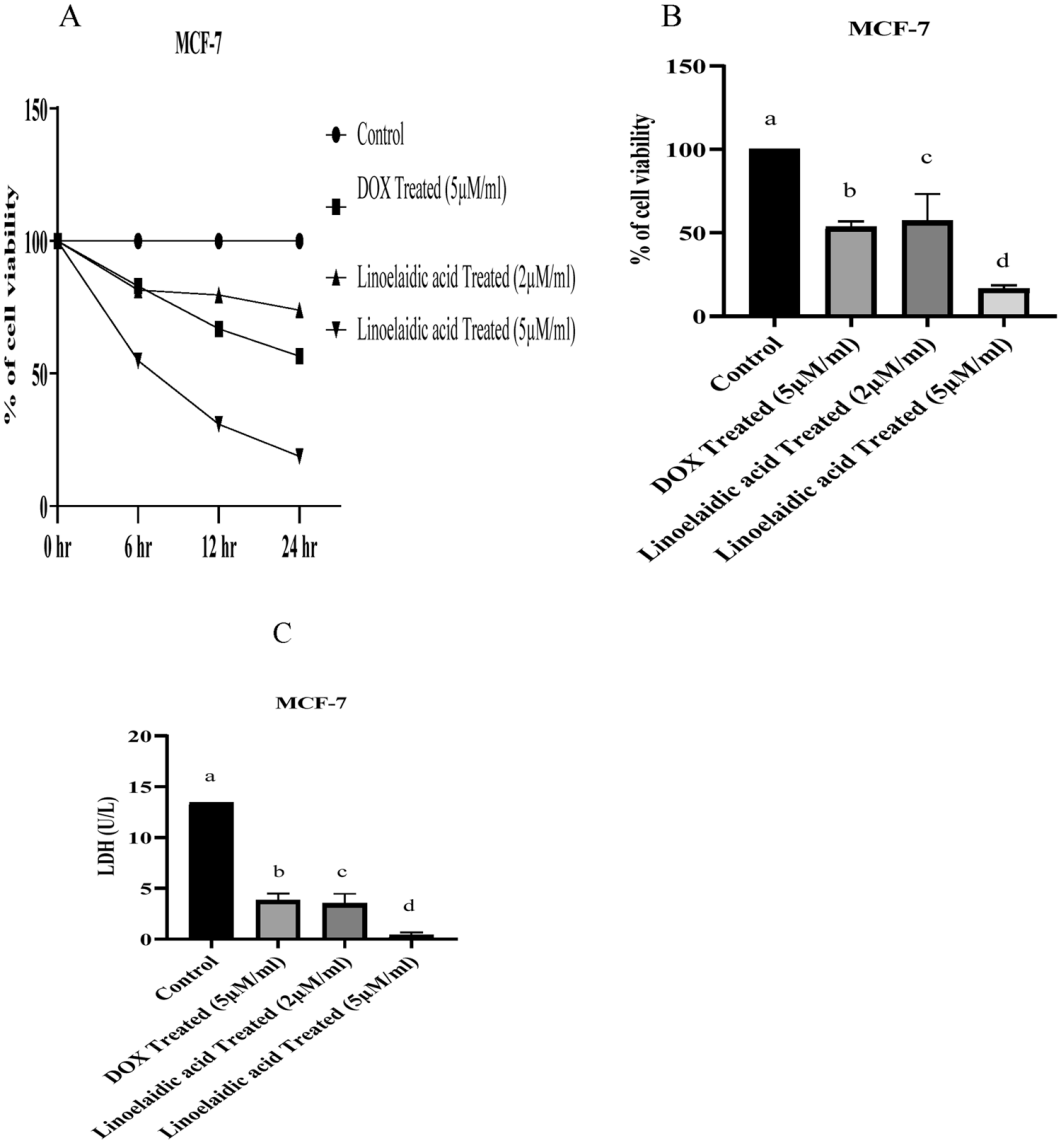
Effect of linoelaidic acid (extracted from Tapra fish) on MCF-7cancer cell line (1 × 10^6^ cells/ml). Cell death rates were measured by the MTT method as described in the materials and methods. Cell viability was measured by the MTT method as described in materials and methods (**A**,**B**). (**C**) Cell cytotoxicity was measured by the LDH method as described in materials and methods; values are expressed as mean ± SEM (n = 3); superscripts indicate significant differences (P < 0.05) compared with the control group.

Preliminary screening of the viability of MCF-7 cells using linoelaidic acid at different doses (2 and 5 μM/ml) compared to doxorubicin treatment at 5 μM/ml concentration. The reduction in cell viability of MCF-7 cells after linoelaidic acid treatment was observed in a time and dose-dependent manner (Fig. 1A,B). The time-dependent study depicted that 100% of the viable cell at starting point of treatment which were significantly (P< 0.05) reduced with the continued treatment up to 24 h. Our results indicated that 68.28%, 33.9%, and 21.25% viability of MCF-7 cells after 6 h,12 h, and 24 h of linoelaidic acid (5 μM/ml) treatment respectively (Fig. 1A). Hence, linoelaidic acid with 5 µM/ml concentration for 24 h showed a promising effect on the reduction of cell viability of MCF-7 cells in comparison to control cells (without treatment of linoelaidic acid).

It was noteworthy that 41.28% and 78.13% of cell deaths were observed after 2 µM and 5 µM of linoelaidic acid treatment respectively. There was remarkable (by more than 50%) decreased in cell viability after treatment with a 5 μM concentration of linoelaidic acid (Fig. 1B). Based on the obtained result, cell differentiation of the treated groups was reduced in a concentration and time dependent manner in comparison to untreated group.

We therefore, assessed the LDH level which could be used as a diagnostic indicator of tumour metastasis. LDH levels are frequently utilised as markers of tissue injury. Linoelaidic acid caused a dose dependent decreased in LDH levels of 68.35% and 88.28% at 2 and 5 μM/ml concentration, respectively, indicating inhibition of cell proliferation in MCF-7 cells compared to untreated cells (Fig. 1C). However, at higher concentration, linoelaidic acid (5 μM/ml) caused reduction of release in LDH reaching a maximum of 88.28%, confirming further the greater chemosensitivity of MCF-7 cells to linoelaidic acid.

### Linoelaidic acid regulates inflammation in MCF-7 cells

**Figure 2:**
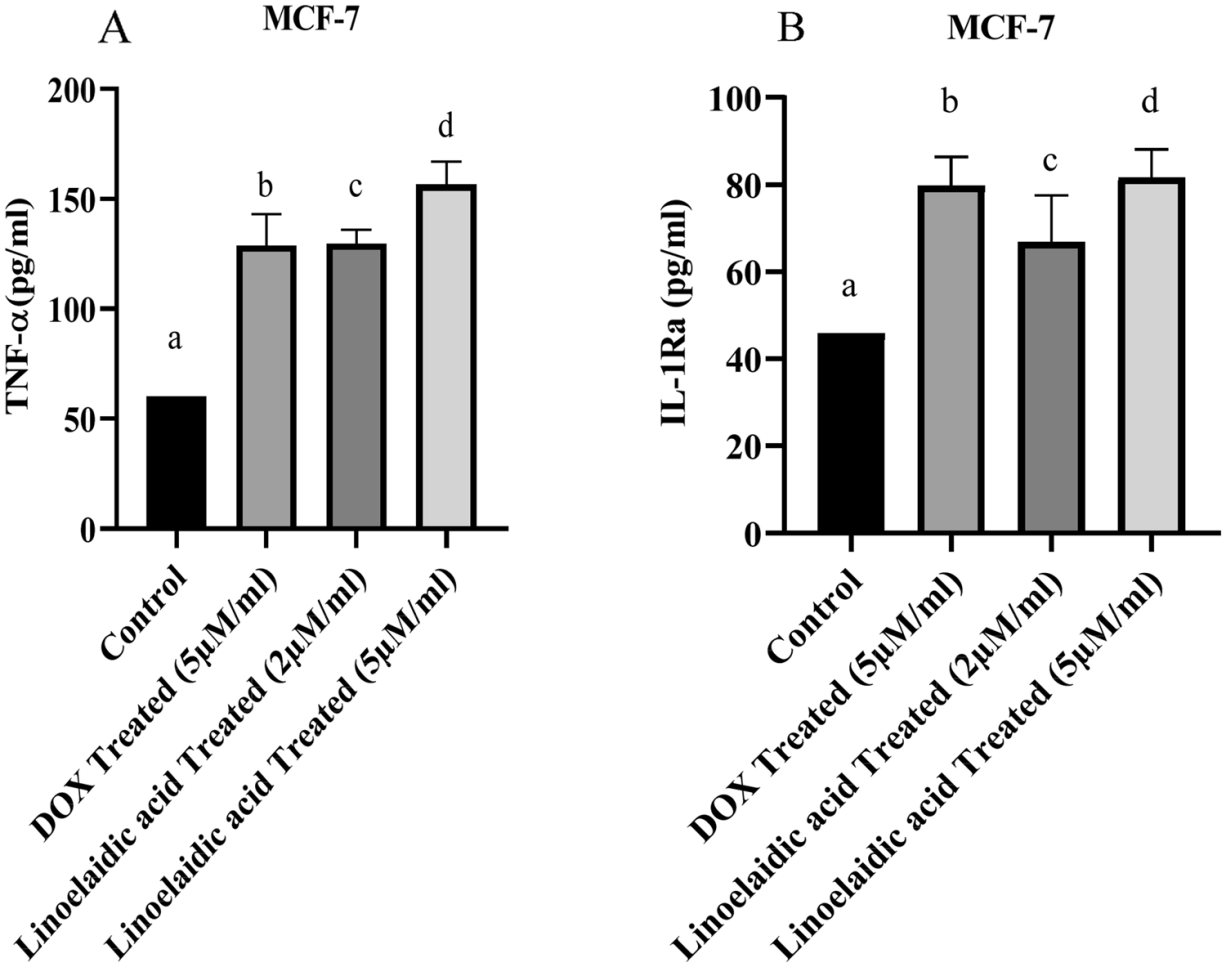
Effect of linoelaidic acid (extracted from Tapra fish) on MCF-7 cancer cell line (1 × 10^6^ cells/ml). Cell inflammation rates and anti-inflammation rates were measured by the ELISA method using TNF-α and IL-1ra as described in materials and methods. Measurement of TNF-α and IL-1ra was expressed by pg/ml. TNF-α/ml values are expressed as mean ± SEM (n = 3); superscripts indicate significant differences (P < 0.05) compared with the control group.

Next, we confirmed the effects of linoelaidic acid on MCF-7 cells by pro-inflammatory cytokine (TNF-α) and anti-inflammatory cytokine (IL-1ra). Inflammation was examined by linoelaidic acid treatment in different doses (2 and 5 μM/ml) on MCF-7 cells in comparison to control cells and DOX treatment (5 μM/ml). A marker of inflammation in cancer cells is the level of TNF-α released into the media. Based on these findings, the levels of TNF-α secreted into cell culture media were enhanced by the treatment of linoelaidic acid in MCF-7 cells (Fig. 2A) and the levels of IL-1ra secretion were also enhanced by the treatment of linoelaidic acid in MCF-7 cells (Fig. 2B). Linoelaidic acid at the dose of 5 μM/ml, can markedly rise in TNF-α and IL-1ra level in breast cancer cells. Our results confirmed a substantial amount of MCF-7 cells were necrosed by the treatment of linoelaidic acid as well as it has an anti-inflammatory role.

### Examination of cellular redox status (NO, GSH, GSSG and MDA level)

**Figure 3:**
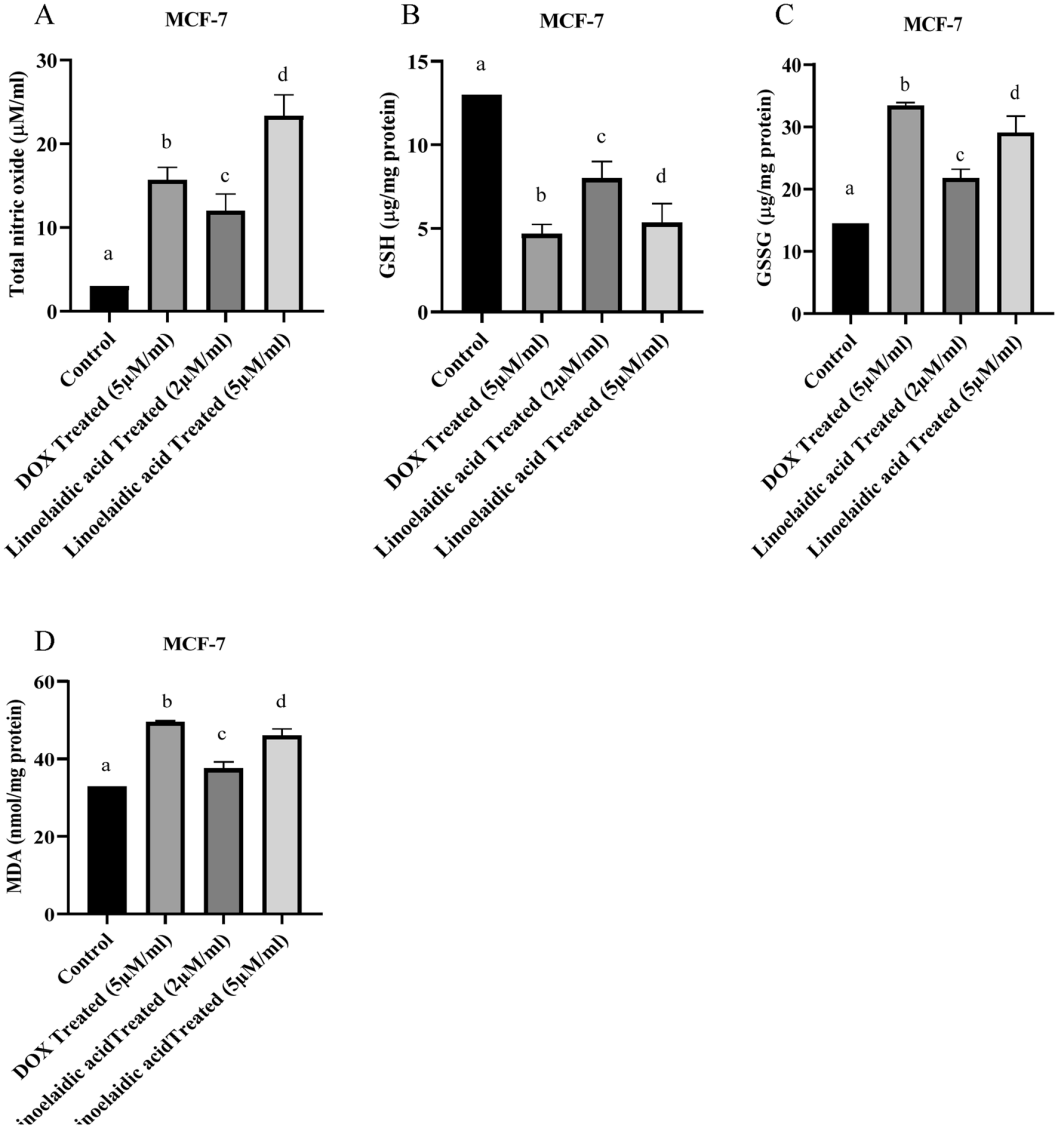
(**A**) Effect of linoelaidic acid (extracted from Tapra fish) on MCF-7cancer cell line (1 × 10^6^ cells/ml). NO was measured and values are expressed as mean ± SEM (n = 3); superscripts indicate significant differences (P < 0.05) compared with the control group. (**B**) Intracellular reduced glutathione (GSH) level of linoelaidic acid-treated, MCF-7 cancer cell line (1 × 10^6^ cells/ml). The level of GSH was expressed as µg of GSH/mg protein. Values are expressed as mean ± SEM (n = 3) of three experiments; superscripts indicate significant differences (P < 0.05) compared with the control group. (**C**) Intracellular oxidized glutathione (GSSG) level of linoelaidic acid-treated, MCF-7 cancer cell line (1 × 10^6^ cells/ml). The level of GSSG was expressed as µg of GSH/mg protein. Values are expressed as mean ± SEM (n = 3) of three experiments; superscripts indicate significant differences (P < 0.05) compared with the control group. (**D**) Intracellular lipid peroxidation (MDA) level of linoelaidic acid-treated, MCF-7 cancer cell line (1 × 10^6^ cells/ml). The level of MDA was expressed as nmol of MDA/mg protein. The values are expressed as mean ± SEM (n = 3) of three experiments; superscripts indicate significant differences (P < 0.05) compared with the control group.

To evaluate oxidative stress, we also investigated nitric oxide, GSH and GSSG levels in linoelaidic acid-treated MCF-7 cells. Nitric oxide is a well-known regulator of vascular smooth muscle tone; it suppresses platelet activation, modifies apoptosis, and regulates inflammatory cell aggregation and activation at low concentrations. In contrast, peroxynitrite (ONOO), which is extremely cytotoxic, can be created when NO reacts with superoxide anion (O^2^). NO level was extremely increased in oxidative stress. Nitric oxide was markedly increased in the MCF-7 cells with the treatment of linoelaidic acid up to 82.16 and 98.35% respectively (Fig. 3A). There was a significant (P < 0.05), dose-dependent increased in NO levels with increasing linoelaidic acid concentrations. High level of NO can enhance stress in cancer cells indicating inhibition of the progression of cancer through suppressing tumor growth, angiogenesis, migration, and metastasis processes in breast carcinoma.

The total GSH content was significantly higher in untreated MCF-7 cells. Linoelaidic acid treatment caused the GSH level in MCF-7 cells to drop by 11.06 and 29.35% considerably (P < 0.05) in comparison to the control (Fig. 3B). On the other hand, Doxorubicin also remarkably reduced the GSH levels in MCF-7 cells. Similar to the earlier experiments, the effect of a higher dose (5 μM/ml) of linoelaidic acid was found to be better than the lower dose (2 µM/ml) of linoelaidic acid.

When compared to the control group of MCF-7 cells, the linoelaidic acid-treated MCF-7 group's GSSG level was 33.25 and 79.6% higher, which was elevated considerably (P < 0.05) (Fig. 3C). At a dose of 5 μM/ml, the level of GSSG in DOX-treated MCF-7 cells was raised more than 50% in comparison to the untreated cells. After treating MCF-7 cells with 5 μM dose of linoelaidic acid, we noticed significant increase in the GSSG level, which shows an active GSH redox cycle.

Treatment with linoelaidic acid affects not only oxidative stress but lipid peroxidation as well. Malondialdehyde is one of many low molecular weight end-products of lipid hydroperoxide breakdown and is frequently evaluated as an indication of lipid peroxidation. MDA has a strong correlation with a decline in antioxidant properties. Malondialdehyde levels were found to be significantly higher in MCF-7 cells treated with DOX and linoelaidic acid in comparison to control cells. The level of MDA was significantly enhanced after treatment of linoelaidic acid at the dose of 2 and 5 μM/ml up to 26.39 and 44.57% respectively (Fig. 3D). The level of MDA increased significantly (P < 0.05) with increasing linoelaidic acid concentration indicate oxidation of lipids by free radicals and it is one of the main manifestations of oxidative damage in tissues and cells.

### Effect of linoelaidic acid treatment on gene expression of inflammatory and apoptotic markers

**Figure 4:**
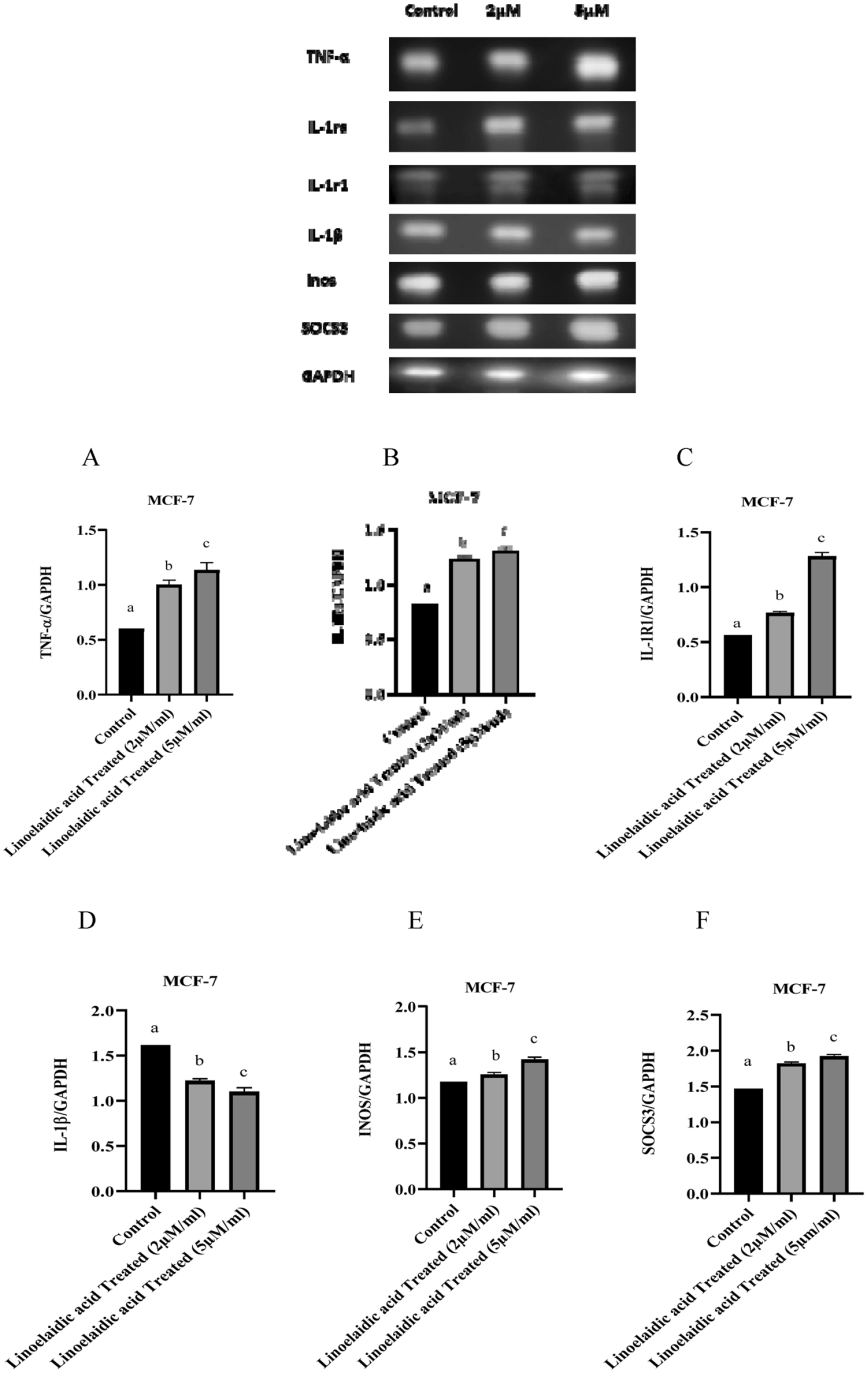
Different gene expression studies through semi q-PCR of linoelaidic acid-treated, MCF-7 cancer cell line (1 × 10^6^ cells/ml). Results were normalized with respect to GAPDH (**A**–**F**). Triplicate values were presented as mean ± SEM. Statistical analysis was performed by one way ANOVA followed by tukey’s multiple comparison test. Superscripts indicate significant differences (P < 0.05) compared with the control group.

The protective effect of genes in linoelaidic acid treated MCF-7 cells was tested. To determine the ability of linoelaidic acid to influence the expression levels of genes associated with inflammation, MCF-7 cells were subjected with two doses of linoelaidic acid 2 and 5 μM/ml, respectively for 24 h. Subsequently, expressions of mRNA levels of inflammatory genes were determined by PCR followed by agarose gel electrophoresis. All expression levels are normalized to GAPDH expression levels. Figure 4A showed that incubation of MCF-7 with 2 μM/ml linoelaidic acid considerably rise the expression levels of TNF-α by 1.66-fold whereas 5 μM/ml remarkably increased it by 1.88-fold in comparison to untreated viable cells. More MCF-7 cells were necrosed by the treatment of 5 μM/ml linoelaidic acid. Figure 4B indicated that linoelaidic acid at the dose of 2 and 5 μM/ml markedly increase the expression levels of IL-1ra in MCF-7 by 1.5 and 1.6-fold, respectively, contrasted to control MCF-7 cells. Likewise, incubation of MCF-7 with 2 and 5 μM linoelaidic acid increased the expression levels of interleukin 1 receptor type 1(IL-1r1) by 1.38 and 2.28-fold compared with control breast cancer cells (Fig. 4C). Figure 4D indicated that linoelaidic acid dose of 2 and 5 μM/ml significantly decreased the mRNA expression levels of IL-1β in MCF-7 by 1.31 and 1.47-fold, respectively, compared to untreated MCF-7 cells. According to Fig. 4E, 2 μM/ml and 5 μM/ml linoelaidic acid slightly enhanced inducible nitric oxide synthase (INOS) expression levels in MCF-7 cells by 1.06 and 1.2-fold, correspondingly, in respect to untreated cells. Similarly, MCF-7 treated with 2 and 5 μM/ml linoelaidic acid exhibits inconsiderable changes in suppressor of cytokine signaling 3 (SOCS3) expression levels (Fig. 4F). Our result illustrated that linoelaidic acid can induced cell death by upregulating the expression of TNF-α, IL-1R1, INOS, SOCS3, but it can reduce the risk of inflammation by diminishing the mRNA expression levels of IL-1β in treated MCF-7 cells.

### Linoelaidic acid induced death in MCF-7 cells is mediated via p53 and caspase activation

**Figure 5:**
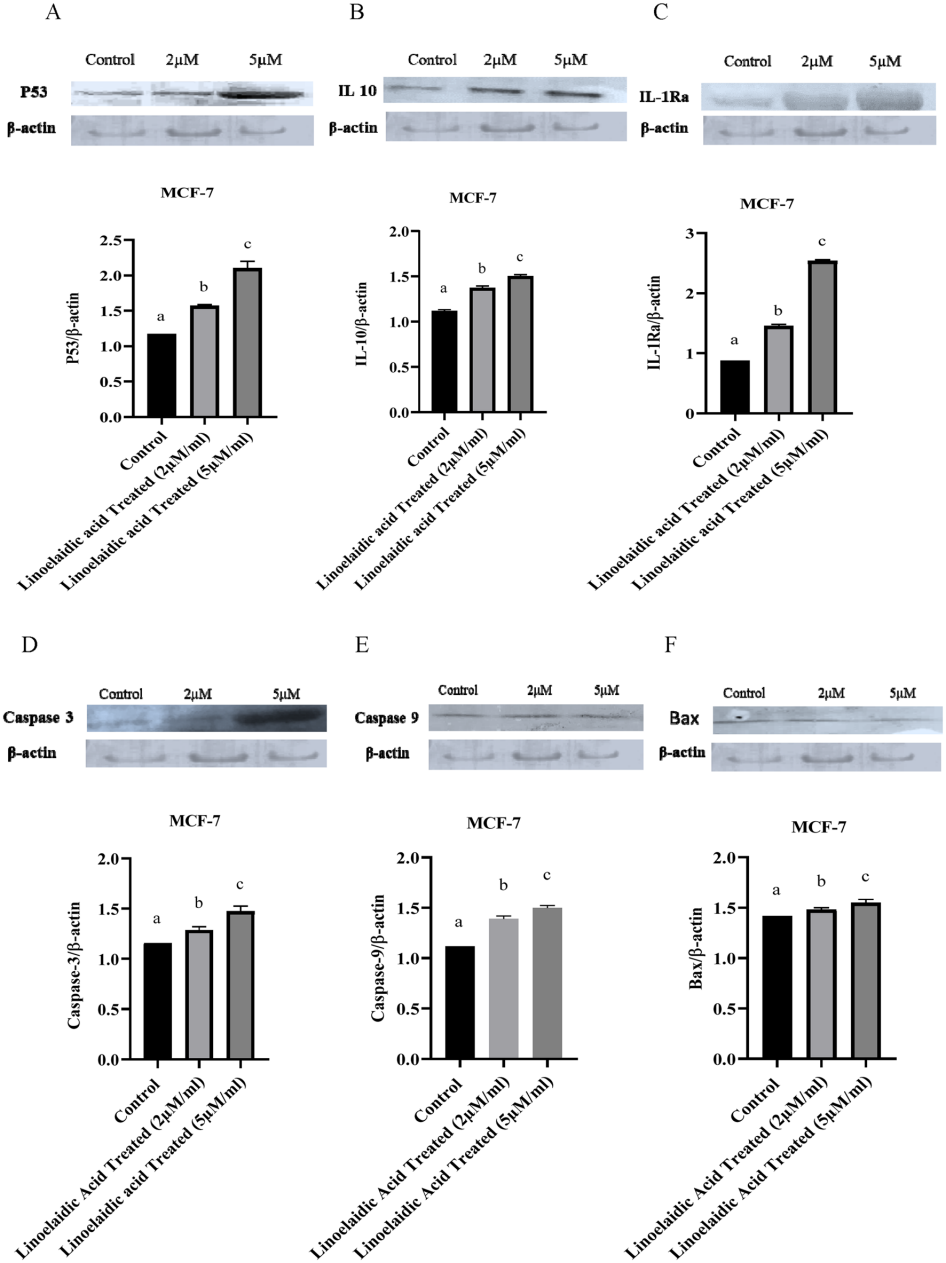
Protein expression study was done on linoelaidic acid treated at two different doses on the MCF-7 cancer cell line (1 × 10^6^ cells/ml) by using Western Blot. Results were normalized to β-actin. Values are expressed as mean ± SEM (n = 3) of three experiments compared with the control group (MCF-7 cells). Statistical analysis was performed by one way ANOVA followed by tukey’s multiple comparison test.

To further investigate the mechanism regarding apoptosis Immunoblotting of p53, IL-10, IL-1Ra, caspase 3, caspase 9, and Bax were assessed. Western blot findings revealed that the expression of the p53 protein, Bax, caspase 3, caspase 9 considerably enhanced as well as the increased expression of IL-10 and IL-1ra, following linoelaidic acid treatments (Fig. 5). To investigate whether the changes in p53, IL-10, IL-1Ra, caspase 3, caspase 9 and Bax mRNA levels are associated with alteration of protein levels, MCF-7 cells were incubated for 48 h with linoelaidic acid at 2 and 5 μM/ml. As shown in Fig. 5A linoelaidic acid significantly increased the protein levels of p53 in treated MCF-7 by approximately 1.34 and 1.79-folds in contrast to control cells, after adjusted to β-actin expression levels. However, the expression of p53 slightly increased in 2 μM linoelaidic acid group. Treatment with linoelaidic acid to MCF-7 cells resulted increased in IL-10 protein levels in comparison to control group (Fig. 5B). Similar to mRNA results, Fig. 5C shows that 2 and 5 μM/ml linoelaidic acid considerably increased the protein levels of IL-1ra in MCF-7 by approximately 1.67 and 2.93-folds, respectively compared to untreated MCF-7 cells after normalization to β-actin levels. In MCF-7 cells, linoelaidic acid significantly increased the expression of caspase 3 by 1.11 and 1.27-fold at the dose of 2 and 5 μM/ml in comparison to untreated MCF-7 cells (Fig. 5D). This figure illustrated that caspase 3 protein expression increased by 1.14-fold at the dose of 5 μM/ml compared to 2 μM/ml treatment of linoelaidic acid. Caspase 9 Similar to this, MCF-7 cells treated with 2 and 5 μM linoelaidic acid exhibited considerably higher levels of caspase 9 expressions by 1.24 and 1.34-fold than untreated cells (Fig. 5E). Caspase 9 protein expression increased by 1.07-fold at the dose of 5 μM/ml compared to 2 μM/ml treatment of linoelaidic acid. Figure 5D,E demonstrate how linoelaidic acid caused caspase 3 and caspase 9 to cleave, resulting in a marked increase in their active forms. We also investigated the effect of linoelaidic acid treatment on gene expression of apoptotic marker. Bax is a pro-apoptotic member of Bcl-2 family. Our data proved that linoelaidic acid promotes Bax overexpression in MCF-7 cell lines (Fig. 5F). Overexpression of Bax is frequently associated to cytotoxicity by inducing the opening of the mitochondrial voltage-dependent anion channel. Treatment with linoelaidic acid at the dose of 5 μM/ml, led to the overexpression of p53 protein and anti-inflammatory cytokines (IL-1ra and IL-10) on MCF-7 cells compared to untreated breast cancer cells. Our finding confirmed that MCF-7 cells treated with linoelaidic acid can induce apoptosis process as well as it protects our body from inflammation which can prevent cell proliferation. Linoelaidic acid dramatically raised the levels of caspase 3 and caspase 9 protein expression, demonstrating that it can induce apoptosis in MCF-7 breast cancer cells. The maximum activation rates of caspase 3 and caspase 9 were noticed in highest concentration of linoelaidic acid treated MCF-7 cells i.e., 5 μM/ml.

### Morphological changes in MCF-7 cells treated with linoelaidic acid

**Figure 6:**
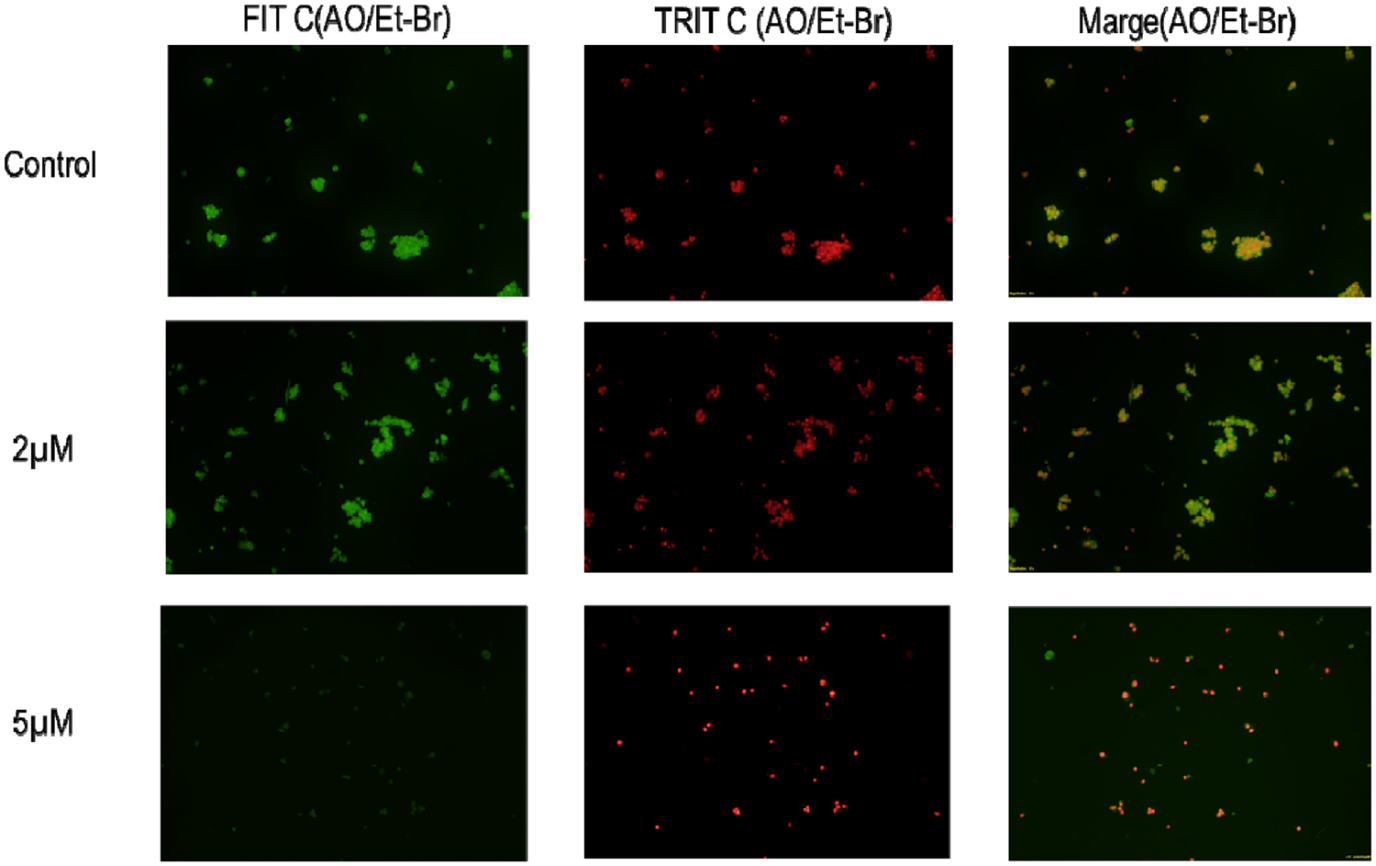
Detection of apoptosis by acridine orange and ethidium bromide (AO-EtBr) staining method in linoelaidic acid-treated at two different doses on MCF-7 cancer cells (1 × 10^6^ cells/ml) by using a fluorescence microscope (Olympus). Values are expressed as mean ± SEM (n = 3) of three experiments compared with the control group (MCF-7 cells).

To compare the morphological alterations in cell membranes of treated and control cells dual acridine orange/ethidium bromide (AO/EB) fluorescent staining can be used that are connected to apoptosis. The MCF-7 cells were subjected to linoelaidic acid at doses of 2 and 5 μM/ml to assess the induction of apoptosis, and after being stained with AO/EB, fluorescence microscopy was used. As shown in Fig. 6 control group did not show any discernible signs of apoptosis. The experimental group showed early-stage apoptotic cells, identified by crescent-shaped or granular yellow-green AO nuclear staining. There were more early-stage apoptotic cells with 5 μM/ml linoelaidic acid treatments. Asymmetrically localised, dense late-stage apoptotic cells with orange nuclear EB staining were also observed. Necrotic cells grew larger and exhibited patchy orange-red fluorescence at their borders seen in linoelaidic acid treated MCF-7 cells. The outcomes demonstrated the apoptotic properties of linoelaidic acid.

### Linoelaidic acid induces intracellular ROS generation in MCF-7 cells

**Figure 7:**
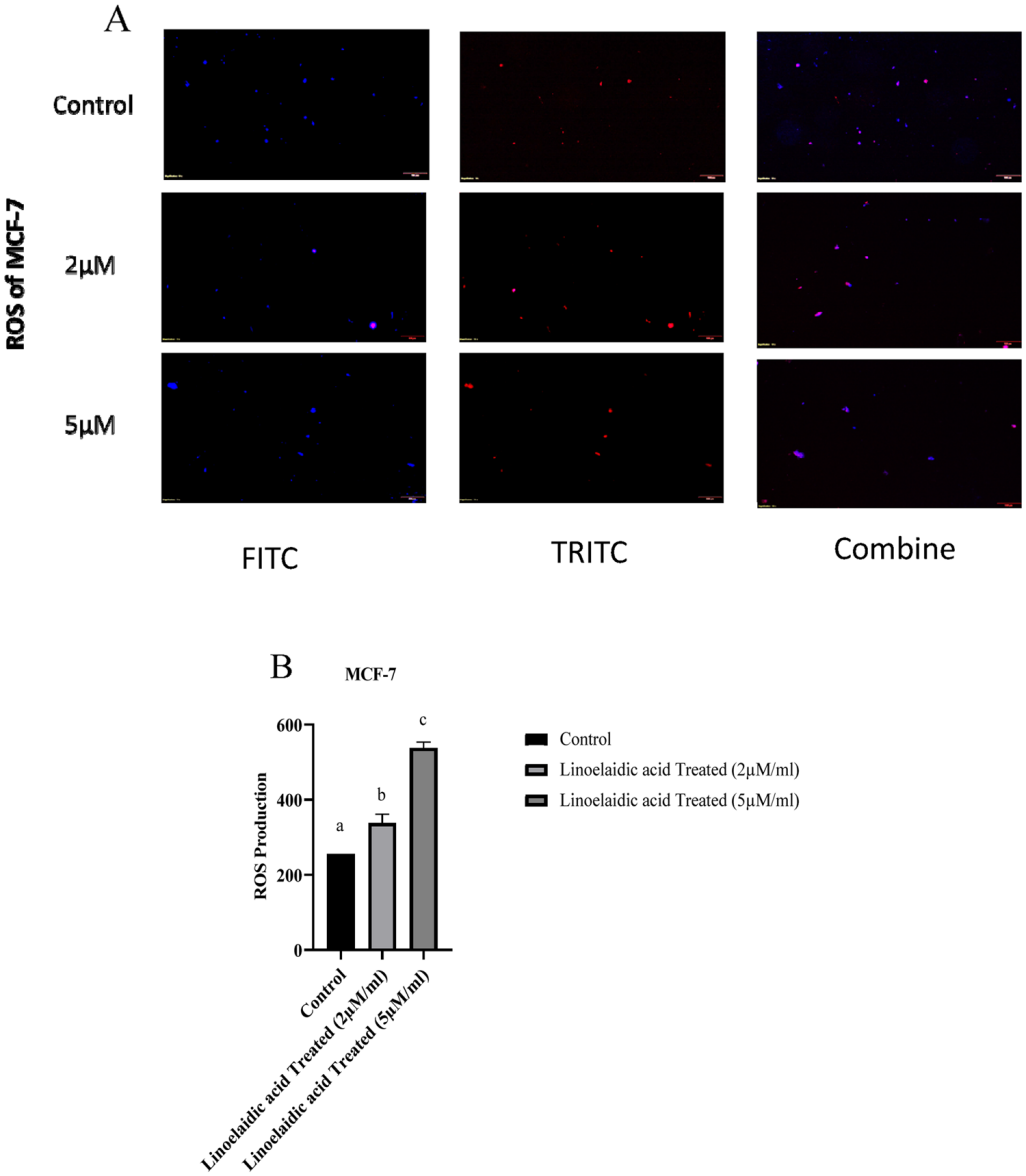
(**A**) Intracellular reactive oxygen species measurement was done from linoelaidic acid treated MCF-7 cells. The levels of ROS were considered as DCF fluorescence intensity and recorded by Fluorescence microscopic images here. ROS generation was observed under a fluorescence microscope at × 40 magnification. (**B**) Measurement of ROS production in Multimode microplate reader at 480 and 530 nm.

Recent research has demonstrated that chemotherapeutic drugs can cause cancer cells to undergo apoptosis by increasing ROS production or decreasing ROS scavenging ability. In consideration of this, we analyzed the stress oxidative components in the currently tested molecule i.e., linoelaidic acid. ROS formation was analyzed on the basis of the study of DCF signals. Fluorescence intensity generated by H_2_DCFDA was increased in a dose-dependent manner in linoelaidic acid treated MCF-7 cells and was much greater than that of the untreated MCF-7 cells (Fig. 7A). At the dose of 5 μM/ml, linoelaidic acid triggered substantial generation of ROS in cancer cells. These results demonstrated a dose-dependent increase in the degree of apoptosis. Our research revealed that linoelaidic acid massively increased cellular ROS levels, which in turn caused apoptosis. It appears that the situation of oxidative damage is connected to the rise in cell death. Based on the results, we put forth the hypothesis that linoelaidic acid might cause the human breast cancer cell line (MCF-7) to undergo oxidative stress-induced apoptosis.

Numerous studies have demonstrated that excessive intracellular ROS generation had a propensity to damage DNA and result in DNA strand breaks, which led to cell apoptosis. We attempted to clarify the role of ROS in the development of linoelaidic acid-induced apoptosis in this investigation. Figure 7B shows the differential effects of linoelaidic acid at the dose of 2 and 5 μM on ROS level in MCF-7 cells. In MCF-7 cells, increases of levels of ROS-sensitive fluorescent probe (CM-H2DCFDA) were observed in linoelaidic acid-treated cells compared to untreated cells. As an outcome, it was found that linoelaidic acid seemed to induce apoptosis in MCF-7 cells.

### Linoelaidic acid promotes apoptosis in MCF-7 cells

**Figure 8:**
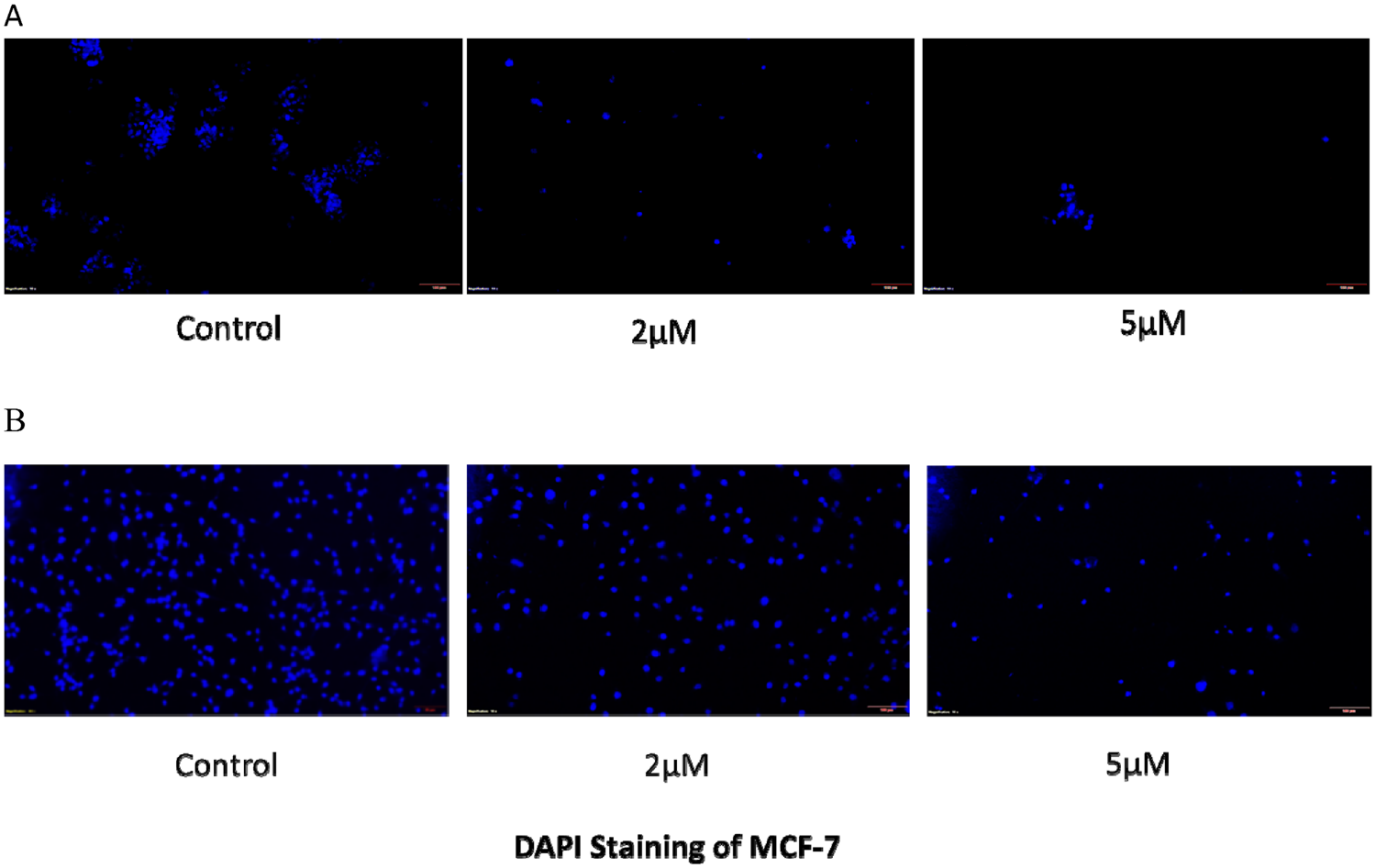
Nuclear morphology of cancer cells was seen by DAPI staining of linoelaidic acid-treated MCF-7 cells and recorded by Fluorescence microscopic images here at × 40 magnification.

In the current study, MCF-7 cells were exposed to various doses of linoelaidic acid (2 and 5 μM/ml) for 24 h in order to investigate its impact on the apoptosis of breast cancer cells. We conducted morphological analysis of the breast cancer cells. Using DAPI staining, nuclear morphological changes in MCF-7 cells were seen under a fluorescent microscope. Condensed and fragmented chromatin, a sign of apoptotic cell death, was visible after staining. As shown in Fig. 8 at the dose of 5 μM, linoelaidic acid can decreased number of live cells by inducing apoptosis. However, the MCF-7 cells that were treated with 2 μM linoelaidic acid showed negligible alterations from the untreated group. The findings showed that when cells were treated with linoelaidic acid (2 and 5 μM/ml) for 24 h, in contrast to the untreated cells, the number of dead cells significantly increased.

## Discussion

Therapeutic strategies applied in cancer treatment involve surgery, radio and chemotherapy which have numerous side effects. In this scenario, natural compounds like flavonoids, isothiocyanates, sulfides, thiols, phenol- derived, alkaloids, marine fish oil derived omega-3, omega-6 fatty acids and other substances extracted from plants as well as from different sea fish emerge as possible anticancer agents^24^. In our previous study, we reported that marine Tapra fish contained linoelaidic acid^5^. In this study, we showed that linoelaidic acid has strong anticancer activities in a cultured human breast cancer cell line (MCF-7) through a list of actions as suppression of cell viability, altering redox balance, inducing apoptosis via caspase activation. Mechanistically linoelaidic acid induced cell death occurred via increased ROS generation and caspase activation^25^. From the MTT/cell viability assay, we observed that the linoelaidic acid exhibited promising anticancer effect with no or minimum toxicity on peripheral blood lymphocytes (PBLs). MCF-7 cell killing activity was shown higher after 24 h treatment of 2 and 5 µM/ml linoelaidic acid compared to untreated cells. The reduction in cell viability of MCF-7 cells after linoelaidic acid treatment was shown in a dose and time-dependent manner (Fig. 1B). The time and concentration-dependent study illustrated that, linoelaidic acid with 5 µM/ml concentration for 24 h showed a promising (P < 0.05) outcome on the reduction of viability of MCF-7 cells. The MTT assay, which is based on the enzymatic conversion of MTT to insoluble formazan in live cells, was used in the current investigation to assess the cytotoxic effect of linoelaidic acid against MCF-7 cells. MTT results revealed that linoelaidic acid had a substantial inhibitory effect on MCF-7 cells following a 24-h treatment with 5 µM/ml, indicating that these cells might be more sensitive to this molecule. This result is in good agreement with the findings of the other investigations, where clearly linoelaidic acid effectively inhibits the proliferation of breast cancer cells^26^.

TNF-α is a well-known inflammatory marker and has a crucial role in MCF-7 cell death^27^. In contrast, IL-1ra being the anti-inflammatory cellular marker^28^. IL‐1ra possesses a tumor‐suppressing effect by preventing IL1 from attaching to its target receptor^29^. Linoelaidic acid treatment significantly (P < 0.05) increased TNF-α and IL-1ra level in MCF-7 cells (Fig. 2A,B). These reinstated the inflammatory environment followed by inducing the cancer cell death after treatment. Several reports proved that TNF-α promoted nitric oxide production via INOS-2 activation^30^. Our results also indicated the enhanced NO production in linoelaidic acid-treated MCF-7 cells (Fig. 3A). Hence, linoelaidic acid-induced TNF-α may promote the nitrite generation in MCF-7 that played a crucial role in cancer cell growth arrest. GSH levels were decreased significantly (P < 0.05) in MCF-7 cells after treatment with two different doses of linoelaidic acid which might help in protection against different cellular peroxides, free radicals, and toxic compounds in the cell^31^. Out of these two doses, 5 µM of linoelaidic acid was found capable to reduce the GSH level in MCF-7 cells (Fig. 3B). Besides that, GSSG levels were significantly (P < 0.05) increased after linoelaidic acid exposure to MCF-7 cancer cells (Fig. 3C). It is well established that GSSG is toxic to the cell and is counter balanced by the GSH level. The decreased level of GSH and increased GSSG level in MCF-7 confirmed the cellular stress after linoelaidic acid treatment. These results promptly indicated that linoelaidic acid modulated the redox balance leading to cell death in MCF-7 and consequently prevent cancer. As a consequence of the alteration in redox balance, MCF-7 cells were experienced with lipid peroxidation (MDA level), the important cellular marker of an oxidative stress response leading to the cell membrane damage^32^. Significantly elevated level of MDA in linoelaidic acid-treated MCF-7 cells was supported this as well as an indicator of MCF-7 cell damage (Fig. 3D).

Next the stability of reference genes was validated under different experimental series with the dose-dependent exposure with linoelaidic acid (2 µM/ml and 5 µM/ml) on MCF-7 cells. The expression of TNF-α and IL-1ra mRNA expressions were examined as inflammatory and anti-inflammatory markers since they were expected to show the variation in expression. In well accordance to the ELISA results (Fig. 2), mRNA expression of inflammatory markers (TNF-α and INOS) was increased whereas IL-1β was decreased and anti-inflammatory markers (IL-1r1, IL-1ra, and SOCS3) were positively expressed in linoelaidic acid-treated MCF-7 cells (Fig. 4). These findings suggested that linoelaidic acid could suppress inflammation but might upregulate cell death by inhibiting tumorigenesis.

TP53/p53, a tumour suppressor gene, is mutated in around 50% of all human malignancies. In addition to its role in tumour suppression, p53 also has a significant impact on how both malignant and non-transformed cells react to various anticancer therapies, especially those that result in DNA damage. Cell cycle arrest, cell senescence, DNA repair, metabolic adaptability, and cell death are just a few of the biological activities that p53 reportedly controls by directly regulating about 500 target genes in a homotetrameric transcription factor^33^. The primary mechanism by which p53 suppresses tumour formation was thought to be the triggering of apoptosis in nascent neoplastic cells^34^. To examine the effect of linoelaidic acid on p53 expression, we performed the Western blotting and the expression level of p53 was found to be increased in a dose-dependent manner (Fig. 5). In this context, increased p53 after linoelaidic acid exposure restricted the MCF-7 cell proliferation via modulating genes related to the cell cycle and apoptosis^35^. Our results evidenced that linoelaidic acid mediated apoptosis was initiated via the expression of mitochondrial Bax and p53 among the different biological origins. Caspases are synthesized as pro-caspases, an inactive form which cleaved into their active form during apoptosis. Hence, determination of caspase activity is considered a gold standard for apoptosis detection^36, 37^. Several caspases, may be activated during apoptosis, however, activation of caspase-3 is crucial, being considered a hall-mark of apoptosis^38^. The biochemical and morphological alterations connected to apoptosis are brought about by activated caspase-9, which mobilises a series of effector caspases, such as caspase-3. Our results indicated that linoelaidic acid exerted a cytotoxic effect on MCF-7 cells in a dose dependent manner.

Duo AO/Et-Br fluorescent staining detects the basic morphological changes in apoptotic cells and identifies the distinction between normal cells, early and late apoptotic cells, and necrotic cells. We observed that AO penetrated normal and early apoptotic cells with intact membranes. It produced fluorescing green illumination in MCF-7 cells without treatment. Et-Br only entered cells with damaged membranes in late apoptotic and dead cells, emitting orange-red fluorescence when bound to concentrated DNA fragments or apoptotic bodies^39^, while, dual AO/Et-Br staining can detect mild DNA injuries^40^. Linoelaidic acid exposure to MCF-7 cells with two different dosages, 2 μM/ml and 5 μM/ml, showed apoptotic signature. In corroborating with previous experiments, 5 μM of linoelaidic acid-induced apoptosis in a greater number of MCF-7 cells compared with 2 μM/ml (Fig. 6).

There are numerous cell signalling mechanisms in cancer cells, especially those associated with ROS generation and scavenging. Although ROS are by products of regular metabolism, their effects on cells depend on their concentration. In intracellular signalling, ROS work as “redox messengers” at low concentrations, but at high concentrations, they can trigger oxidative alteration of macromolecules, which can impair cellular functions and hasten apoptosis^41^. Cancer cells, in contrast to healthy cells, have increased metabolic activity, which results in a persistently higher amount of ROS and supports a long-lasting pro-oxidative atmosphere. However, cancer cells can endure high amounts of metabolic ROS by modifying their anti-oxidative systems and up-regulating pro-survival processes in order to adapt to the oxidative environment^42^. By this way, active molecules that induce increasing in intracellular ROS can assume an outstanding importance in cancer therapy. It was verified that the mechanisms by which linoelaidic acid promotes apoptosis involve^43^, at least in part, the ROS increase^44^. It was proved that linoelaidic acid at (5 μM/ml) significantly increased ROS generation (Fig. 7A,B). Thus, it was conferred that excess production of ROS in cancer cell leads to cell death ultimately prevent abnormal cell proliferation and differentiation. Furthermore, as evidenced by DAPI staining, cells treated with linoelaidic acid exhibit typical apoptotic alterations with chromatin condensation and nuclear disintegration (Fig. 8).

## Conclusion

In conclusion, linoelaidic acid treatment at its selected dose caused a decrease in the viability of MCF-7 cells accompanied by an increase in ROS levels. Treatment with linoelaidic acid caused a decrease in intracellular ATP levels indicating impairment in the mitochondrial function as confirmed by dissipation in the membrane potential and cyt-c release in culture media; disruption of plasma membrane integrity was confirmed by LDH release with no change in cell motility or invasiveness. Additionally, linoelaidic acid induced apoptosis in MCF-7 cells through activating the capacity to fragment DNA. Our findings reveal that linoelaidic acid can give rise to cell death via apoptosis pathway in MCF-7 cells. Elucidation of the key players underlying the sensitivity to linoelaidic acid and mechanistic differences between normal and cancer cells to linoelaidic acid requires further investigations. Further in vivo study is needed before clinical application.

### Supplementary Information

Supplementary Information 1.

Supplementary Information 2.

## Data Availability

The datasets generated during and/or analysed during the current study are available from the corresponding author on reasonable request.
