# Supplementary material for: Anti-cancer potentiality of linoelaidic acid isolated from marine Tapra fish oil (Ophisthopterus tardoore) via ROS generation and caspase activation on MCF-7 cell line
Source: Sci Rep. 2023 Aug 29;13:14125. doi: 10.1038/s41598-023-34885-3 (PMC10465529; doi:10.1038/s41598-023-34885-3)
Supplement: Supplementary file 1 — Supplementary Information 1. [file 41598_2023_34885_MOESM1_ESM.pdf]

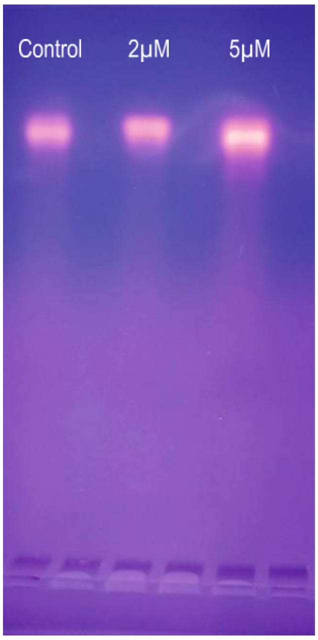

TNF- α (Figure-S1)

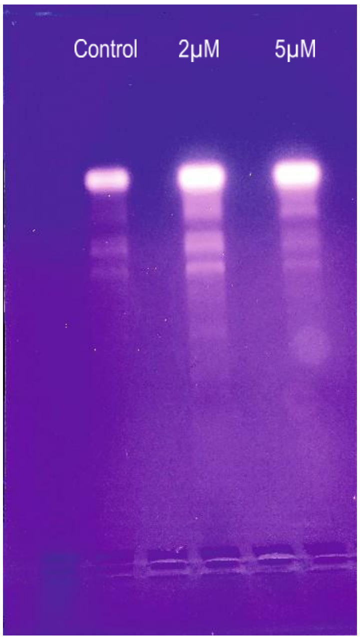

IL-1Ra (Figure-S2)

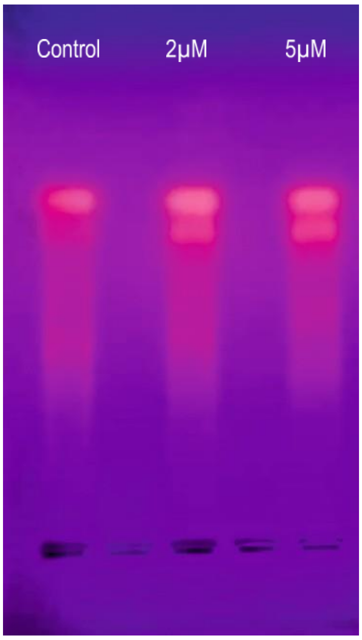

IL-1R1 (Figure-S3)

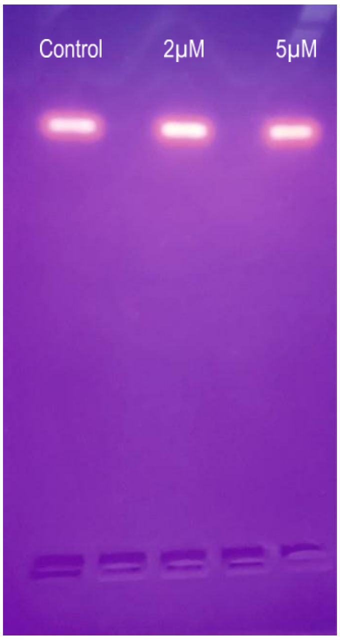

IL-1β (Figure-S4)

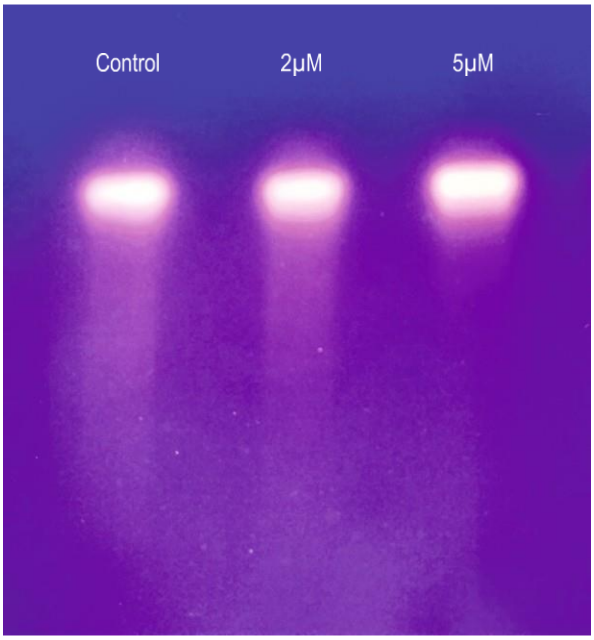

INOS (Figure-S5)

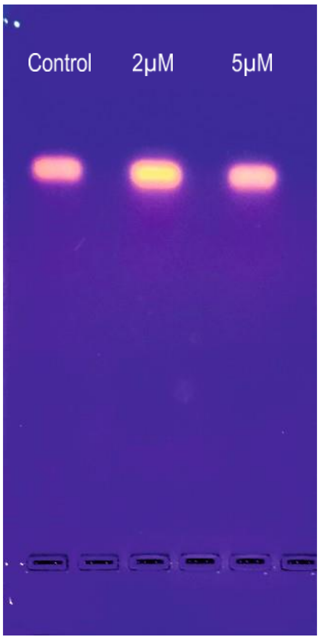

SOCS3 (Figure-S6)

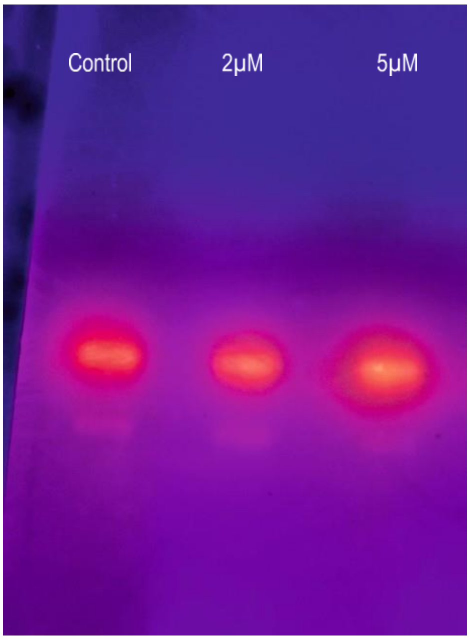

GAPDH (Figure-S7)

Figure (S1-S7). Different gene expression studies through semi-q-PCR of linoelaidic acid-treated, MCF-7 cancer cell line (1×10^6 cells/ml) at two different doses (2μM/ml & 5μM/ml). Results were normalized with respect to GAPDH (S7). (S1) TNF-α, (S2) IL-1Ra, (S3) IL-1R1, (S4) IL-1β, (S5) INOS, (S6) SOCS3.

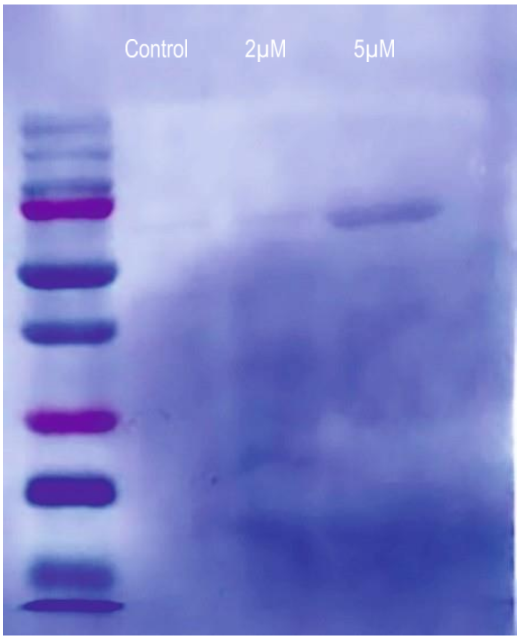

P53 (Figure-S8)

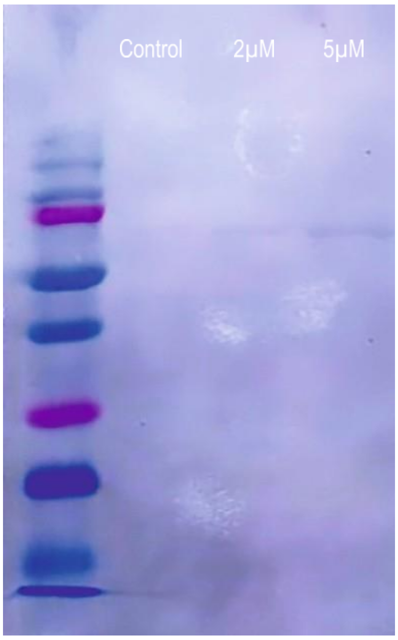

IL-10 (Figure-S9)

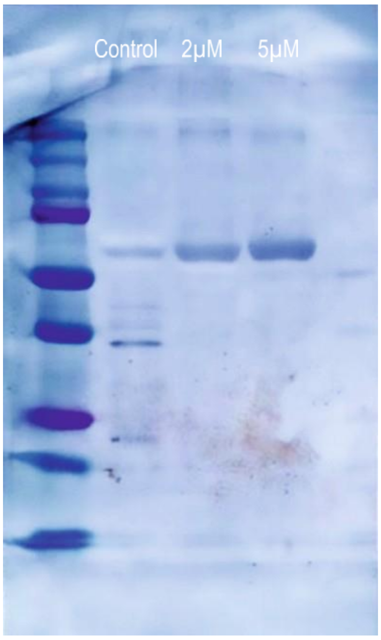

IL-1Ra (Figure-S10)

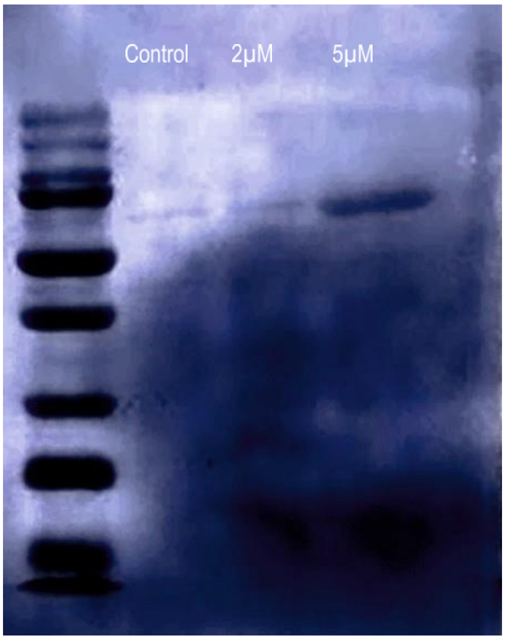

Caspase-3 (Figure-S11)

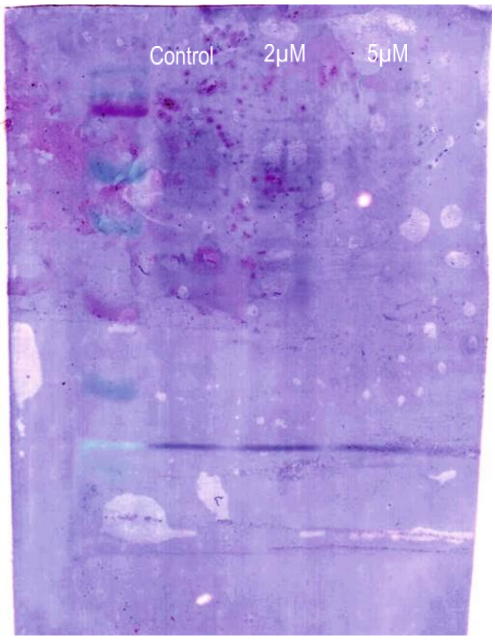

Caspase-9 (Figure-S12)

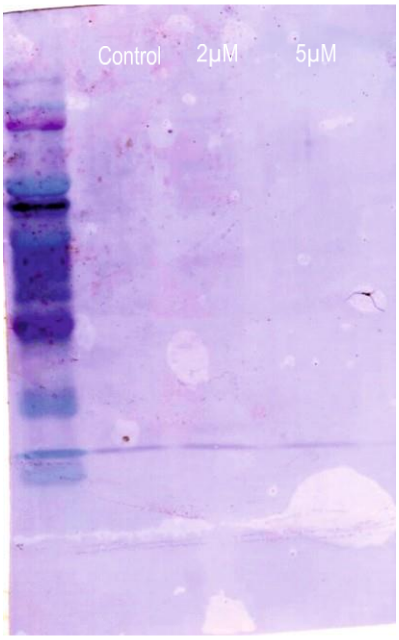

BAX (Figure-S13)

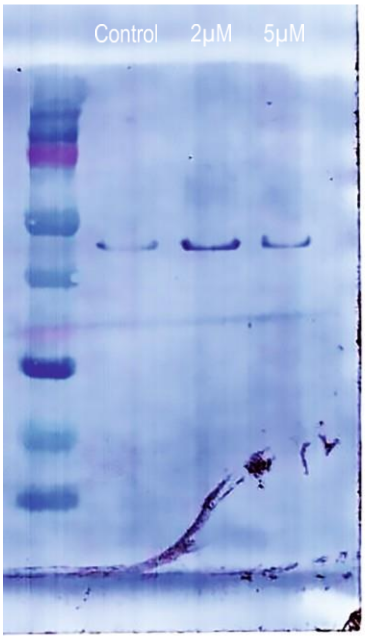

Beta-actin (Figure-S14)

Figure (S8-S14). A protein expression study was done on linoelaidic acid treated at two different doses (2μM/ml & 5μM/ml) on the MCF-7 cancer cell line (1×10^6 cells/ml) by using Western Blot. Results were normalized to (S14) β-actin. (S8) P53, (S9) IL-10, (S10) IL-1Ra, (S11) Caspase 3, (S12) Caspase 9, (S13) Bax.

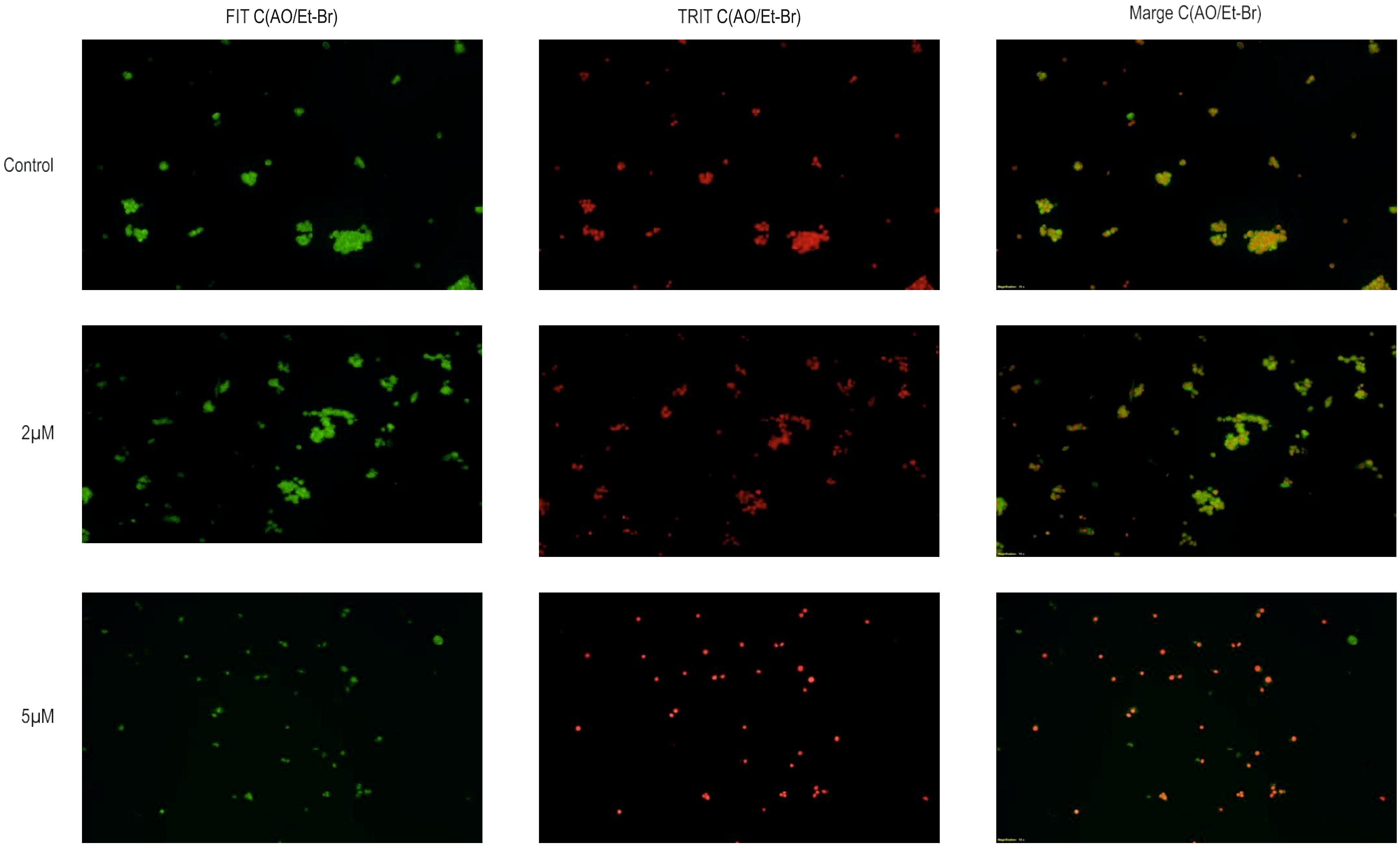

Figure S15. Detection of apoptosis by acridine orange and ethidium bromide (AO-EtBr) staining method in linoelaidic acid-treated at two different doses (2μM/ml & 5μM/ml) on MCF-7 cancer cells (1×10^6 cells/ml) by using a fluorescence microscope (Olympus). Values are expressed as mean ± SEM (n=3) of three experiments compared with the control group (MCF-7 cells).

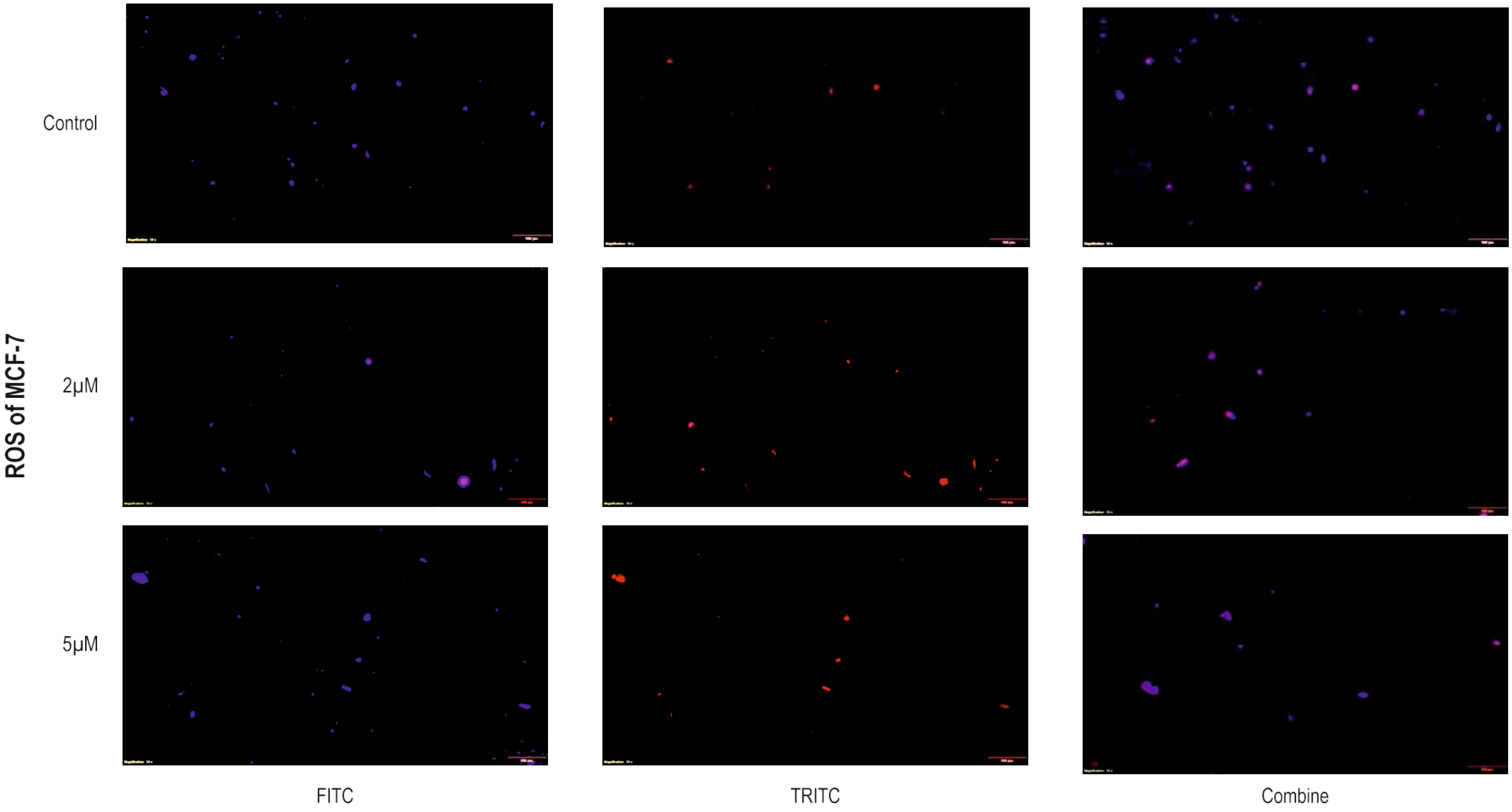

Figure S16. Intracellular reactive oxygen species measurement was done from linoelaidic acid treated MCF-7 cells. The levels of ROS were considered as DCF fluorescence intensity and recorded by Fluorescence microscopic images here. ROS generation was observed under a fluorescence microscope at 40x magnification.

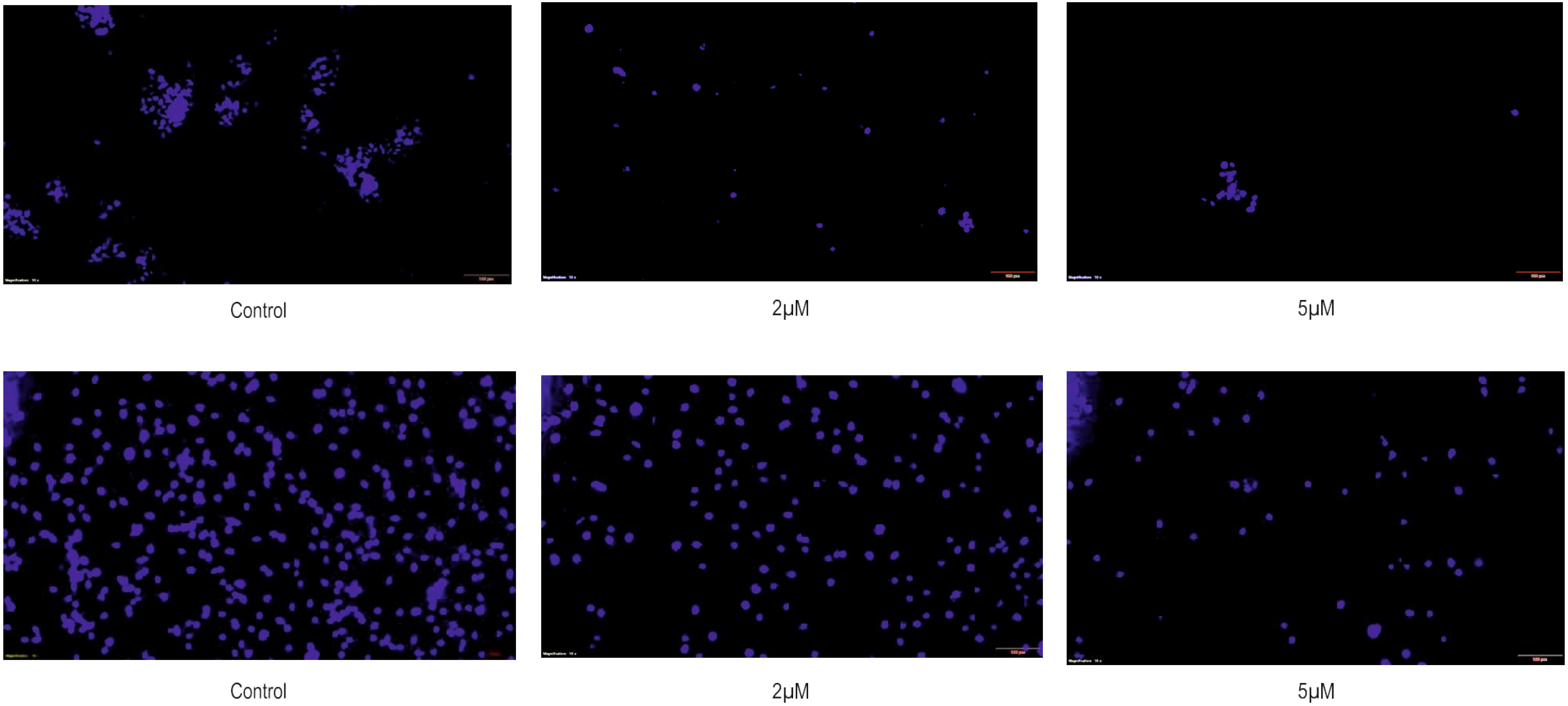

DAPI Staining of MCF-7

Figure S17. The nuclear morphology of cancer cells was seen by DAPI staining of linoelaidic acid-treated MCF-7 cells and recorded by Fluorescence microscopic images here at 40x magnification.
