# Supplementary material for: Anti-cancer potentiality of linoelaidic acid isolated from marine Tapra fish oil (Ophisthopterus tardoore) via ROS generation and caspase activation on MCF-7 cell line
Source: Sci Rep. 2023 Aug 29;13:14125. doi: 10.1038/s41598-023-34885-3 (PMC10465529; doi:10.1038/s41598-023-34885-3)
Supplement: Supplementary file 2 — Supplementary Information 2. [file 41598_2023_34885_MOESM2_ESM.docx]

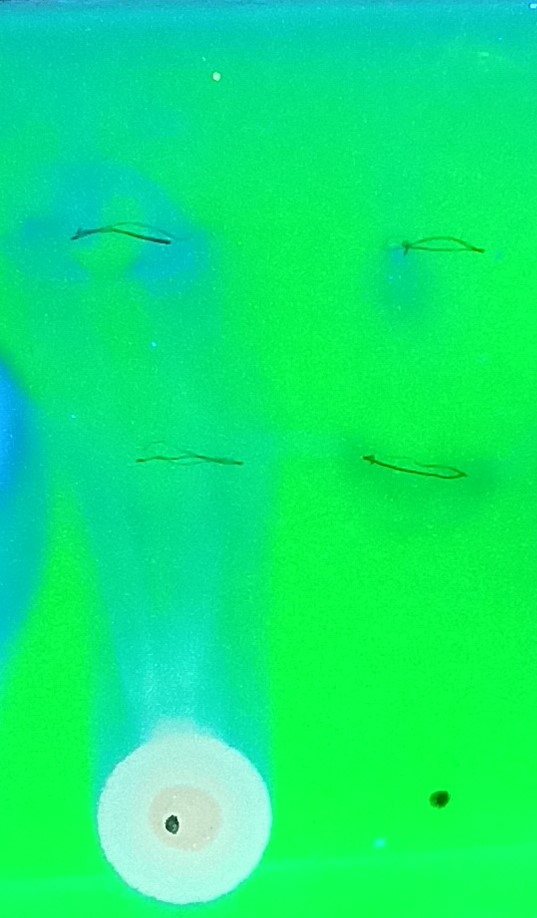


Tapra Fish Oil Std Linoelaidic Acid

FIGURE 1 Presence of linoelaidic acid on extracted **O. tardoore** fish oil using Thin Layer Chromatography.
